# S100A family is a group of immune markers associated with poor prognosis and immune cell infiltration in hepatocellular carcinoma

**DOI:** 10.1186/s12885-023-11127-3

**Published:** 2023-07-07

**Authors:** Yuchen Qi, Yujing Zhang, Jianwen Li, Mengting Cai, Bo Zhang, Zhangtao Yu, Yuhang Li, Junkai Huang, Xu Chen, Yinghui Song, Sulai Liu

**Affiliations:** 1 Department of Hepatobiliary Surgery, Central Laboratory, Hunan Provincial People’s Hospital, The First Affiliated Hospital of Hunan Normal University, Changsha, Hunan Province 410005 China; 2 Department of Cardiology, Xiangdong Hospital Affiliated to Hunan Normal University, Liling, Hunan Province 412200 China; 3 Central Laboratory of Hunan Provincial People’s Hospital, The First Affiliated Hospital of Hunan Normal University, Changsha, Hunan Province 410005 China; 4 Key Laboratory of Molecular Epidemiology of Hunan Province, School of Medicine, Hunan Normal University, Changsha, China; 5 Department of Nuclear Medicine, Hunan Provincial People’s Hospital, The First Affiliated Hospital of Hunan Normal University, Changsha, Hunan Province 410005 China; 6 Department of Minimally Invasive Surgery, The Second People’s Hospital of Hunan Province, Changsha, Hunan Province 410005 China

**Keywords:** Cancer, HCC, S100A family, S100A10, Biomarker

## Abstract

**Background:**

Hepatocellular carcinoma (HCC) is one of the most common human cancers with poor prognosis in the world. HCC has become the second leading cause of cancer-related death in China. It is urgent to identify novel biomarker and valid target to effectively diagnose, treat or predict the prognosis of HCC. It has been reported that S100A family is closely related to cell proliferation and migration of different cancers. However, the values of S100As in HCC remain to be further analyzed.

**Methods:**

We investigated the transcriptional and translational expression of S100As, as well as the value of this family in HCC patients from the various databases.

**Results:**

S100A10 was most relevant to HCC.

**Conclusions:**

The results from HCC patients’ tissues and different cells also confirmed the role of S100A10 in HCC. Furthermore, we proved that S100A10 could influenced the cell proliferation of HCC cells via ANXA2/Akt/mTOR pathway. However, it would appear that the relationship between S100A10 and HCC is complex and requires more research.

**Supplementary Information:**

The online version contains supplementary material available at 10.1186/s12885-023-11127-3.

## Introduction

Hepatocellular carcinoma (HCC) is the most frequent liver primary cancer and represents the sixth malignant neoplasm and the third cause of cancer-associated deaths worldwide [1, 2]. HCC can be treated by surgical resection, interventional therapy, targeted therapy, immunotherapy and liver transplantation, etc., but only surgical treatment for the early stage of HCC is known as radical treatment [3, 4]. However, most patients with HCC are usually diagnosed as III or IV stage at the first visit, therefore they are not eligible for radical treatment [5]. Despite the widespread use of local interventional therapy combined with targeted therapies or immunotherapy for advanced HCC in the last decade, the overall prognosis of HCC remains poor [6]. Therefore, it is crucial to identify prognostic biomarkers and therapeutic targets associated with HCC.

The S100A protein is a calcium-binding protein that has only been detected in vertebrates, and its coding gene *S100A* is localized on chromosome 1q21 [7]. It has been reported that S100A proteins are a family of small acidic proteins (10–12 kDa) that are profoundly involved in regulating transcription, differentiation, survival, proliferation and motility of cells [8]. Recently, experimental and clinical researches illustrate that the expression of some S100A proteins in various tumors is extremely associated with poor prognosis and tumor development [9–11]. Therefore, they are potentially important tumor diagnostic and prognostic biomarkers and therapeutic targets. For instance, previous studies reported that S100A2 and S100A10 were the negative biomarker for pancreatic cancer (PC), while S100A4 was profoundly involved in the chemoresistance and survival in are negative prognostic biomarkers in PC cells [12–14]. Besides, it has been reported that S100A families related to various tumor cell proliferation partly dependent on PI3K/AKT signaling pathway [15]. However, the relationship between S100A families and proliferation or apoptosis of HCC( Over-expression of S100A2 in pancreatic cancer correlates with progression and poor prognosis. S100A10, a novel biomarker in pancreatic ductal adenocarcinoma. S100A4 contributes to the suppression of BNIP3 expression, chemoresistance, and inhibition of apoptosis in pancreatic cancer), Whether the expression change of this family member has a certain correlation with the proliferation and apoptosis of hepatoma cells is not clear. In addition, the role of PI3K/AKT signaling pathway in the proliferation of HCC regulated by S100A needs to be further explored.

In the study, various public databases and experimental analysis were used, we extended the research field of HCC with the purpose to determine the values of S1001A in HCC. Moreover, the role of S100A10 was in the cell growth and apoptosis of HCC and the underlying mechanism was also determined. The findings in the present study indicated the relationship between S100As and HCC development, especially S100A10. It also provided a potential mechanism of S100A10 in promoting HCC proliferation and development via PI3K/mTOR pathway.

## Materials and methods

### Identification of *S100As* mRNA status

The expression of *S100As* mRNA levels were obtained from The Cancer Genome Atlas (TCGA) database(http://cancergenome.nih.gov/)that is a comprehensive database with coordination program of gene expression and clinical information. The database is a landmark cancer genomics program that covers more than 20,000 primary cancer tissues. It also provides researchers with detailed clinical and pathological information of 33 human cancers. We downloaded the high-throughput RNA-sequencing data in Hepatocellular carcinoma (LIHC) and the clinical information data. The transcript expression levels were estimated using the fragments per kilobase per million fragments mapped (FPKM) method in HTSeq. After R package (ggplot2[3.3.6], stats[4.2.1] and car) is analyzed by R software (4.2.1), Visualize the data with the ggplot2 package.

### Identification of S100As protein status

In the present study, in addition to analyze the mRNA levels of *S100As*, the expression of S100As proteins were supplied by the data of Clinical Proteomic Tumor Analysis Consortium (CPTAC) Confirmatory/Discovery dataset for LIHC. The subtypes, based on proteome, could be inspected in exterior tumor proteomic datasets. In our study, the protein expressions of S100As between LIHC samples and corresponding normal samples were performed, the statically remarkable difference was considered when *P* < 0.001.

### Analysis of survival data and drawing of ROC curve

Gene Expression Profiling Interactive Analysis (Gepia2) website (http://gepia2.cancer-pku.cn/) was applied to analyze the survival data related to different cancer patients in the Genotype-Tissue Expression (GTEx) database (www.gtexportal.org). The influence of *S100As* gene expression level on the prognosis of each tumor was analyzed. Besides, the survival data of HCC patients were obtained from the TCGA database. According to the median of *S100As* mRNA expression, all HCC patients were divided into *S100As* mRNA high expression group and *S100As* mRNA low expression group. Finally, the Kaplan-Meier survival curve was drawn by the survminer package and the survival package to analyze the effect of the expression level of *S100As* gene on the clinical prognosis of HCC patients. Furthermore, the results from the TCGA database were verified again through the Kaplan-Meier Plotter website. The Kaplan-Meier Plotter website (www.kmplot.com/) is able to assess the impact of 54k genes (mRNA, miRNA, protein) on survival of 21 cancer types including HCC. The website data comes from Gene Expression Omnibus (GEO), European Genome-phenome Archive (EGA), and TCGA. Then, the clinical diagnostic effects of S100A10 and AFP were compared by the pROC package, and the ROC curve was drawn by the ggplot2 package.

### Analysis of protein-protein interaction network

String website (https://string-db.org) is a database to predict protein-protein interactions (including at least 6 K million proteins). The protein-protein interactions include direct (physical) and indirect (functional) associations. By inputting *S100A* gene into the search column, we can directly obtain The Protein-protein Interaction (PPI) network information map and the interaction correlation coefficient between proteins (the combination score > 0.7 is regarded as close relationship).

### GSEA and GO KEGG analysis

In this study, we analyzed the correlation between S100A10 mRNA expression and all other genes. the cluster Profiler package was applied for GSEA analysis and the org.Hs.eg.db package and cluster Profiler package were used for GO KEGG [16–18] analysis. |ES|>1, P < 0.05, and FDR < 0.25 were considered statistically significant.

### Analysis of tumor-related immune infiltration

The Tumor Immune Estimation Resource Web Server (TIMER) is a comprehensive resource for systematic analysis of immune infiltration in different cancer types. In order to analyze the correlation between the expression of S100A10 and immune infiltration in HCC tissues, first six types of immune cells including B cells, CD4 + T cells, CD8 + T cells, neutrophils, macrophages and dendritic cells were obtained in the TIMER database. Next, the CD8 + T cell, CD4 + T cell, T regulatory cell infiltration and s100a10 expression were calculated through this website. Then, the relationship between each immune cell marker and the S100A10 expression was analyzed. Also, the coefficient value (R) and corresponding P value of the correlation between S100A10 and immune cell marker were obtained from GEPIA.

### Immunohistochemistry (IHC)

The patients whose tissue were used for the present research were provided by informed consent. the HCC tissue samples and adjacent non-tumor tissue samples T were collected at the Hunan Provincial People’s Hospital/The First Affiliated Hospital of Hunan Normal University, Changsha, China, In brief, paraffin-embedded tissues sections were deparaffinized and rehydrated. 3% hydrogen peroxide (H_2_O_2_) was used to block the endogenous peroxidase activity for 30 min at RT, and then the sections were pre-treated with sodium citrate buffer (pH = 6) for 30 min following pressure cooker heat mediated antigen retrieval. The specific primary anti-S100A10 antibody was used to incubate each tissue Sect. (1:1000, 5529; CellSignaling Technology, Danvers, MA, United States) at 4 °C overnight。 Subsequently, the section were incubated with HRP-conjugated secondary antibody at 37 °C for 45 min and stained with 3, 3-diaminobenzidine tetrahydrochloride (DAB) solution. The section was counterstained with haematoxylin, and observed under a microscope.

### Cell culture and transfection

L02, HepG2, Huh7, LM3 and SNU449 cells were cultured in DMEM medium supplemented with 10% fetal bovine serum (FBS, Corning) at 37 °C and 5% CO2. Si-S100A10 (cat. no. sc-36,338, Santa Cruz Biotechnology) and scrambled siRNA (si-NC, cat. no. sc-37,007, Santa Cruz Biotechnology) were applied to silence the expression of S100A10. HCC cells were seeded into 6-well plates at a density of 5 × 10^5^ cells per well. Then, cells were transfected with 100 nM siRNA by using Lipofectamine RNAiMAX reagent (Invitrogen; Thermo Fisher Scientific, Inc.). Approximately 48 h later, the expression of S100A10 was verified by Western blot.

### Overexpression of *S100A10* in HepG2 cells

Transfection was performed in HepG2 cells according to the standard instruction. Briefly, HepG2 cells were seeded in 100-mm cell culture tissue at 2 × 10^6^ cells/well for 6 ~ 8 h. Then the cells transfected with overexpression clone lentiviral of *S100A10* (ov-*e*) and empty vector (EV) from Genechem Co., Ltd. (Shanghai, China). After 5 days of cell expansion and maintence in DMEM medium with 10% FBS, 1.5 µg/ml puromycin was used to select stable HepG2 cell lines expressing S100A10.

### RNA isolation, reverse transcription and QRT-PCR analysis

Total RNA was extracted from all cells according to the kit instructions (Invitrogen, CA, USA). The mRNA expression of S100A10 was detected by by the SYBR Green PCR Master Mix (Takara, Tokyo, Japan). The primer sequences of mRNAs as follow: GAPDH-F: GCACCGTCAAGGCTGAGAAC; GAPDH-R TGGTGAAGACGCCAGTGGA; S100A10-F: AACAAAGGAGGACCTGAGAGTAC; S100A10-R: CTTTGCCATCTCTACACTGGTCC, QRT-PCR was performed following the manufacture’s protocol of the SYBR Green PCR kit (Takara, Tokyo, Japan). Real-Time PCR System was used to quantify the mRNA levels of S100A10 at 95 °C for 30 s, followed by 40 cycles of 95 °C for 5 s and 60 °C for 34 s. ΔCt values were normalized to *ACTB* levels, and the relative expressions of mRNA to the control were calculated by the 2^−ΔΔCT^.

### Protein extraction and western blot

Proteins from frozen tissues and cells were extracted using RIPA buffer with 1% phosphatase inhibitor cocktail and PMSF. The lysates were collected following 30 min lysis on ice. Then the Pierce BCA Protein Assay Kit (Beyotime Institute of Biotechnology, Shanghai, China) were used to detected proteins concentration. The process of western blotting analysis was performed as described previously [12]. 15% sodium dodecyl sulfate-polyacrylamide gel electrophoresis (SDS- onto the nitrocellulose (NC) membranes (PALL, CA, USA). The specific primary antibodies were used to incubate the membrane overnight at 4 °C. Subsequently, the membrane was incubated with horseradish peroxidase-conjugated secondary antibody at 1:5000 dilution for 1 h at RT. The protein bands were observed with Enhanced Chemiluminescence (ECL) Detection Kit(Amersham, Piscataway, NJ, USA). Image J software was used to analyze the relative levels of proteins.

### Cell counting Kit-8 (CCK-8)

For CCK8 assays, cells were seeded into 96-well cell culture tissue at 3000 cell/well. According to the instruction, 10 µl of CCK-8 solution diluted in 100 µl of culture medium replaced the original medium at different time (24, 48, 72, and 96 h) incubating for 1 ~ 2 h. The absorbance was measured by Microplate Reader at a 450-nm wavelength.

### Tumor xenograft assay

Male BALB/c nude mice at 6–8 weeks were obtained from the Shanghai Laboratory Animal Center (Shanghai, China). The animals were housed in standard conditions with light controlled conditions (12-h light/12-h dark cycle) and laboratory food and water ad libitum. Animals were adapted to the environment for 1 week before experiment. The experimental procedures were approved by Hunan Normal University and performed in accordance with the National Institutes of Health Guidelines for the Care and Use of Laboratory Animals. HepG2 and SNU449 cells (2 × 10^6^ cells/mice) were implanted subcutaneously into the flank of nude mice. After tumor formation, tumor volume (mm3) was measured every other day. When the tumors had reached a volume of approximately 600 mm^3^, the mice were euthanized, and the tumors were excised.

### Flow cytometry

Cell apoptosis was assayed by Flow cytometry using fluorescein isothiocyanate (FITC) Annexin V/propidium iodide (PI) kit (Invitrogen, CA, USA) following the manufacturer’s instruction. Briefly, cells were collected and simultaneously stained with FITC-Annexin V (green) and the nonvital dye PI (red), which allowed the discrimination of intact cells (FITC-/PI-), early-to mid-apoptotic (FITC+/PI-) and late apoptotic or necrotic cells (FITC+/PI+).

### Statistical analysis

SPSS 20.0 (SPSS, USA) was used for statistical analysis, and the quantitative data were usually expressed as mean ± standard deviation. Spearman rank correlation analysis was performed to evaluate the correlation of gene expression in tissue array. Kruskal-Wallis test and Dunn’s test were used to analyze the differences in S100A10 expression between clinical variables. Logrank test was used to analyze prognostic effects. Other data were compared by student’s t test or Mann Whitney test. Two-tails of P value less than 0.05 were considered statistically significant.

## Result

### The expression of S100A is higher in some tumor tissues than the corresponding normal tissues

**Fig. 1:**
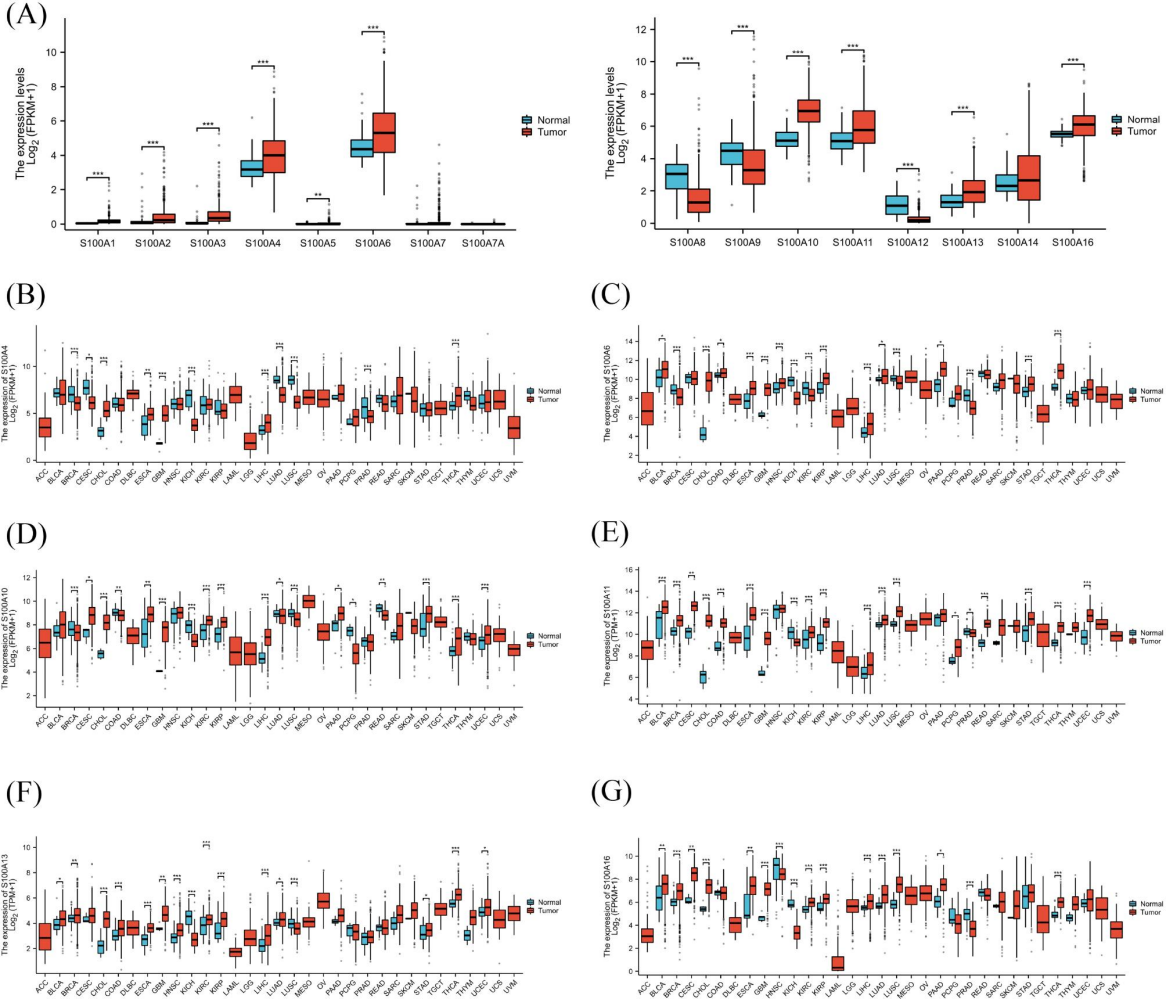
Expression of S100A mRNA in tumor and normal tissue. **(A)** The expression level of S100A mRNA in HCC from TCGA database. **(B)** The expression level of S100A4 mRNA in generalized carcinoma from TCGA database. **(C)** The expression level of S100A6 mRNA in generalized carcinoma from TCGA database. **(D)** The expression level of S100A10 mRNA in generalized carcinoma from TCGA database. **(E)** The expression level of S100A11 mRNA in generalized carcinoma from TCGA database. **(F)** The expression level of S100A13 mRNA in generalized carcinoma from TCGA database. **(G)** The expression level of S100A16 mRNA in generalized carcinoma from TCGA database. Note: *P < 0.05, **P < 0.01, ***P < 0.001. Abbreviations: ACC: Adrenocortical carcinoma; BLCA: Bladder Urothelial Carcinoma; BRCA: Breast invasive carcinoma; CHOL: Cervical and endocervical cancers(CESC), Cholangiocarcinoma; COAD: Colon adenocarcinoma; DLBC: Lymphoid Neoplasm Diffuse Large B-cell Lymphoma; ESCA: Esophageal carcinoma; GBM: Glioblastoma multiforme; HNSC: Head and Neck squamous cell carcinoma; KICH: Kidney Chromophobe; KIRC: Kidney renal clear cell carcinoma; KIRP: Kidney renal papillary cell carcinoma; LAML: Acute Myeloid Leukemia; LGG: Brain Lower Grade Glioma; HCC: Hepatocellular Carcinoma; LUAD: Lung adenocarcinoma; LUSC: Lung squamous cell carcinoma; MESO: Mesothelioma; OV: Ovarian serous cystadenocarcinoma; PAAD: Pancreatic adenocarcinoma; PCPG: Pheochromocytoma and Paraganglioma; PRAD: Prostate adenocarcinoma; READ: Rectum adenocarcinoma; SKCM: Skin Cutaneous Melanoma; STAD: Stomach adenocarcinoma; TGCT: Testicular Germ Cell Tumors; THCA: Thyroid carcinoma; UCEC: Uterine Corpus Endometrial Carcinoma; UCS: Uterine Carcinosarcoma; UVM: Uveal Melanoma

**Fig. 2:**
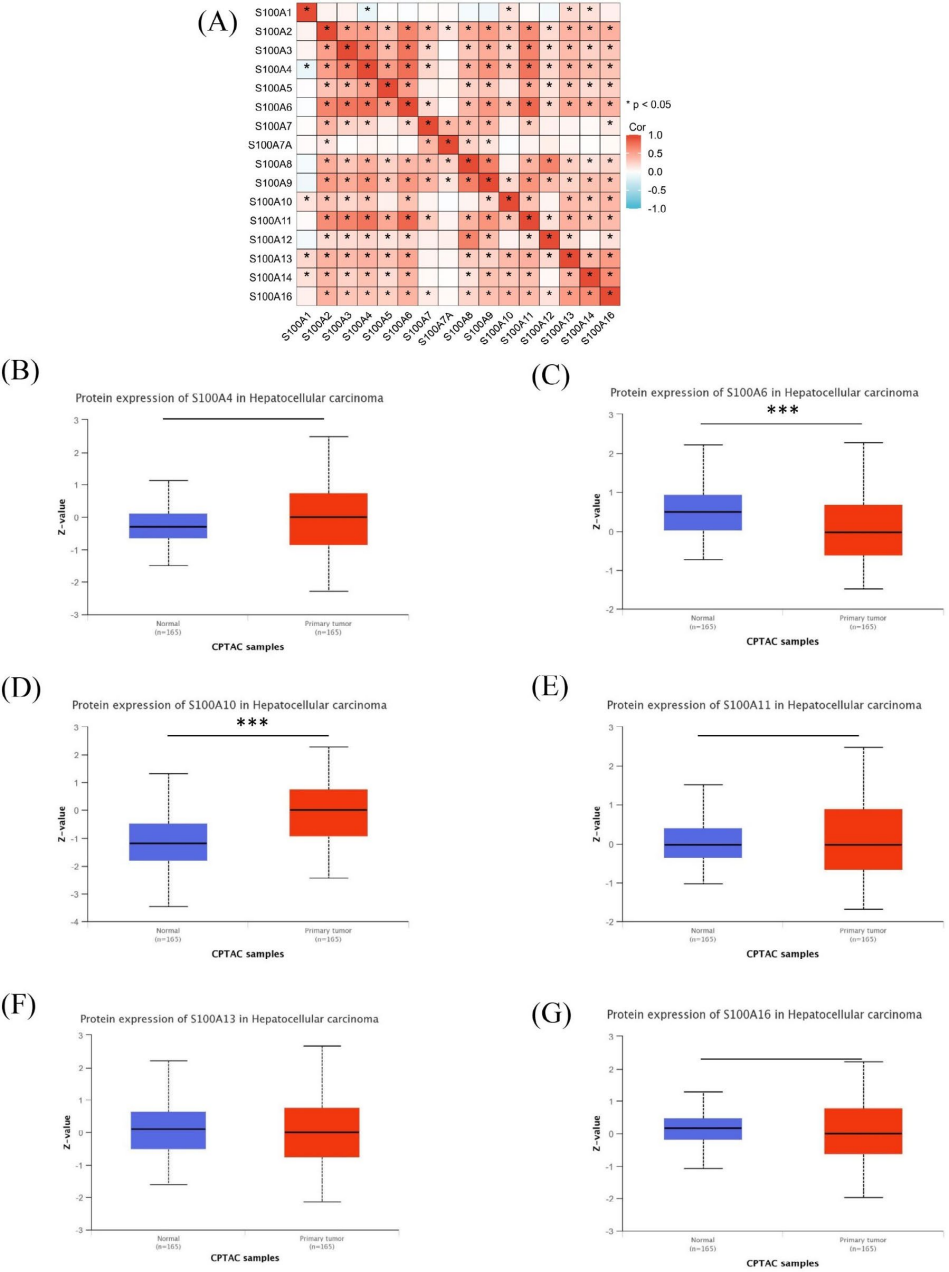
The expression level of S100A protein in HCC. **(A)** Co-expression of S100A in tumors from TCGA database. **(B)** The expression level of S100A4 protein in HCC from CPTAC database. **(C)** The expression level of S100A6 protein in HCC from CPTAC database. **(D)** The expression level of S100A10 protein in HCC from CPTAC database. **(E)** The expression level of S100A11 protein in HCC from CPTAC database. **(F)** The expression level of S100A13 protein in HCC from CPTAC database. **(F)** The expression level of S100A16 protein in HCC from CPTAC database. Note: ***P < 0.001

**Fig. 3:**
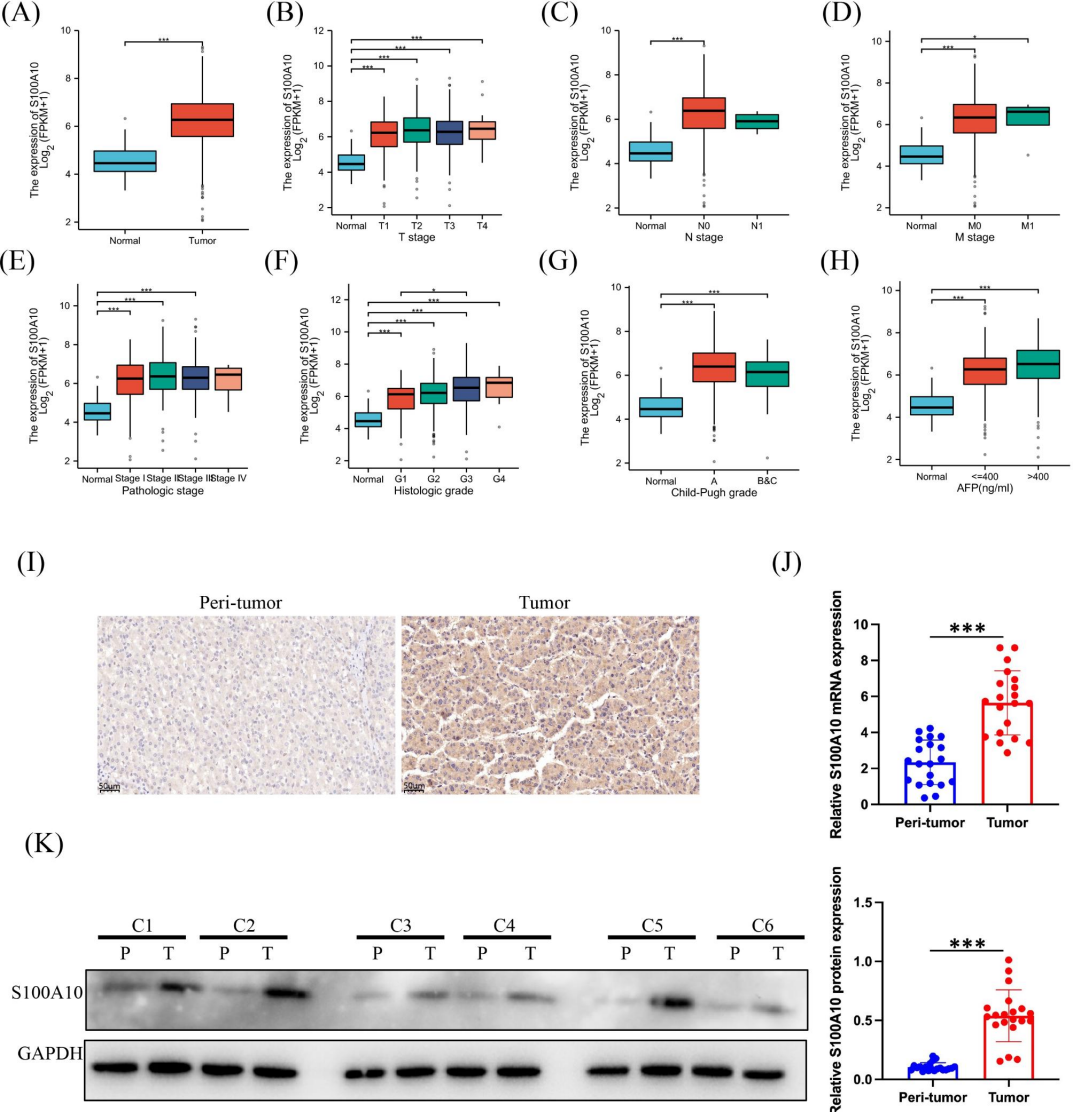
Expression level of S100A10 in HCC. **(A)** mRNA expression of S100A10 was different between HCC and normal tissues. **(B-H)** S100A10 mRNA expression difference in different clinical stages of HCC. **(I)** Immunohistochemistry of HCC tumor and para cancer tissue S100A10. **(J)** Expression of S100A10 mRNA in HCC tumor and para cancer tissues by PCR. **(K)** The expression of S100A10 protein in HCC tumor and para cancer tissue was detected by Western Blot. Note: *P < 0.05, ***P < 0.001

We analyzed the expression of S100A mRNA in HCC tissues and corresponding normal tissues using TCGA database. Meanwhile, the expression of S100A protein in liver cancer tissues and adjacent tissues from CPTAC database. From TCGA database, we collected a total of 424 samples, including RNA sequencing data and detailed clinical prognosis information resources of 374 hepatocellular carcinoma specimens and 50 normal tissue specimens. We found that the expression levels of S100A4, S100A6, S100A10, S100A11, S100A13 and S100A16 in HCC and corresponding normal tissues were high, and the expression levels in tumors were higher than those in normal tissues (Fig. 1A). Further analysis showed that S100A4, S100A6, S100A10, S100A11,S100A13and S100A16 were highly expressed in most tumors (Fig. 1B-G). The expression of S100A members was positively correlated in tumors (Fig. 2A). Meaningful S100A members were screened out by analyzing the mRNA expression level. In order to further understand the protein expression level, we analyzed S100A4, S100A6, S100A10, S100A11, S100A13 and S100A16 through CPTAC database (Fig. 2B-F). The final results showed that there was no significant difference in the expression of S100A4, S100A11, S100A13 and S100A16 between HCC and normal tissues (*P* > 0.1) (Fig. 2B-G). The expression of S100A10 proteins in HCC was significantly higher than that in normal tissues (*P* < 0.01) (Fig. 2D). However, the expression of S100A6 protein in tumors was lower than that in normal tissues (Fig. 2C). S100A6 mRNA expression is higher in tumors, but S100A6 protein expression is lower in tumors, which may be involved in many regulatory mechanisms. According to the above analysis, only mRNA and protein of S100A10 were expressed at a high level in both tumor and normal tissues of HCC, and the expression in tumor tissues was higher than that in normal tissues. Therefore, the next study focused on S100A10. In order to further investigate the expression of S100A10 in HCC, we analyzed the expression of S100A10 in different HCC stages by using TCGA database, although the expression of S100A10 in HCC tumor tissues was higher than that in normal tissues. Interestingly, there was no significant correlation between the expression level of S100A10 and TNM stage, Pathological stage, Histologic grade, child-pugh grade and AFP of HCC (Fig. 3A-H). To verify the reliability of data sources, tumor tissues and para cancer tissues of 40 HCC patients were collected in Hunan Provincial People’s Hospital. Among them, 39 patients showed high expression levels of S100A10 protein in tumor tissues, and 40 patients showed low expression levels of S100A10 protein in para cancer tissues (Fig. 3I). Subsequently, qRT-PCR was used to detect the expression level of *S100A10* mRNA in tumor tissues and para cancer tissues of these 40 patients. Similarly, expression of *S100A10* mRNA in tumor tissues was significantly higher than that in para cancer tissues (*P* < 0.01) (Fig. 3J). The consistent results were obtained by detecting the protein expression level of S100A10 by Western Blot (Fig. 3K). In conclusion, we could basically conclude that S100A10 mRNA and protein are highly expressed in HCC.

### S100A10 has good diagnostic sensitivity in HCC

**Fig. 4:**
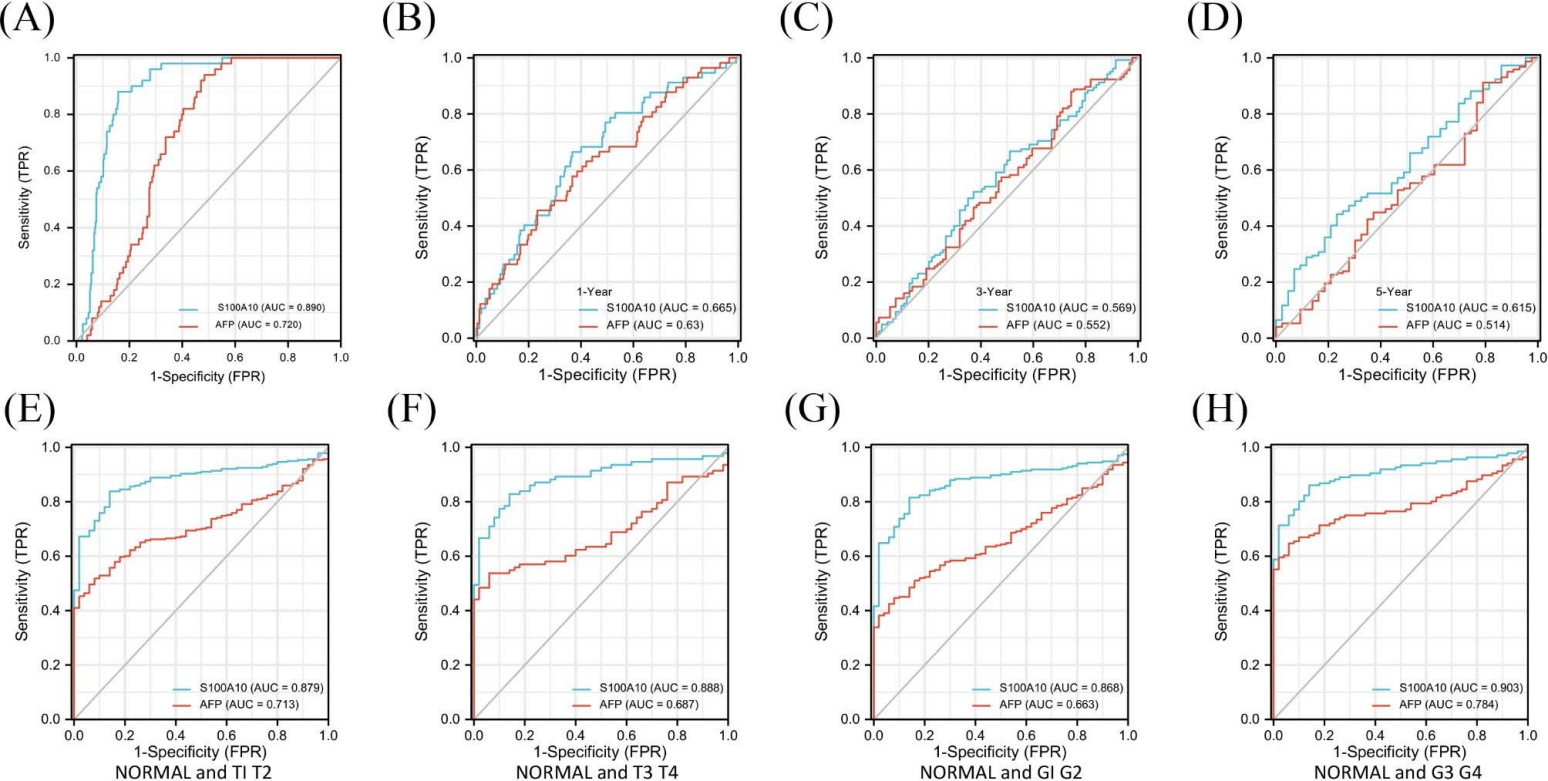
The ROC curve was established by TCGA database. **(A)** Diagnostic efficacy of S100A10 and AFP in HCC. **(B-D)** Differences in the diagnosis of S100A10 and AFP between normal and HCC patients at different timepoint. **(E-F)** Diagnostic efficacy of S100A10 and AFP in different stages of HCC.

In this study, we assessed the diagnostic value of S100A10 in HCC by making ROC curves from the TCGA database. The results showed that the area under the curve (AUG) of S100A10 was 0.890 (Fig. 4A), and the diagnostic performance of S100A10 was better than that of AFP. In addition, we also analyzed the diagnostic value of S100A10 in different HCC stages. The results showed S100A10 AUG = 0.665 at 1 year of tumor progression (Fig. 4B), S100A10 AUG = 0.569 at 3 years of tumor progression (Fig. 4C), and S100A10 AUG = 0.615 at 5 years of tumor progression (Fig. 4D), S100A10 AUG = 0.879 at T1 and T2 stage (Fig. 4E), S100A10 AUG = 0.888 at T3 and T4 stage (Fig. 4F), S100A10 AUG = 0.868 at G1 and G2 stage (Fig. 4G), S100A10 AUG = 0.903 at G3 and G4 (Fig. 4H). All of these data supported that the sensitivity of S100A10 for the diagnosis of HCC is no less than that of AFP.

### The high expression of S100A is associated with poor prognosis

According to the RNA sequencing data of HCC in TCGA database, they were divided into two groups: S100A10 low expression group (n = 187) and S100A10 high expression group (n = 187). The clinical data were grouped according to the following information: tumor status, T stage, N stage, M stage, pathological stage, historical grade, Child-Pugh grade, AFP, race, age, adjacent pathological tissue information, vascular invasion and OS event, and the above information was listed in Table 1.

**Table 1:** Clinical characteristics of the HCC patients

Characteristic	Low expression of S100A10	High expression of S100A10	p
n	187	187	
Tumor status, n (%)			0.026
Tumor free	111 (31.3%)	91 (25.6%)	
With tumor	65 (18.3%)	88 (24.8%)	
T stage, n (%)			0.713
T1	96 (25.9%)	87 (23.5%)	
T2	43 (11.6%)	52 (14%)	
T3	40 (10.8%)	40 (10.8%)	
T4	6 (1.6%)	7 (1.9%)	
N stage, n (%)			0.350
N0	120 (46.5%)	134 (51.9%)	
N1	3 (1.2%)	1 (0.4%)	
M stage, n (%)			0.624
M0	129 (47.4%)	139 (51.1%)	
M1	1 (0.4%)	3 (1.1%)	
Pathologic stage, n (%)			0.820
Stage I	89 (25.4%)	84 (24%)	
Stage II	40 (11.4%)	47 (13.4%)	
Stage III	42 (12%)	43 (12.3%)	
Stage IV	2 (0.6%)	3 (0.9%)	
Histologic grade, n (%)			0.029
G1	35 (9.5%)	20 (5.4%)	
G2	93 (25.2%)	85 (23%)	
G3	52 (14.1%)	72 (19.5%)	
G4	4 (1.1%)	8 (2.2%)	
Child-Pugh grade, n (%)			0.112
A	96 (39.8%)	123 (51%)	
B	13 (5.4%)	8 (3.3%)	
C	1 (0.4%)	0 (0%)	
AFP(ng/ml), n (%)			0.133
<=400	111 (39.6%)	104 (37.1%)	
> 400	26 (9.3%)	39 (13.9%)	
Race, n (%)			0.004
Asian	65 (18%)	95 (26.2%)	
Black or African American	8 (2.2%)	9 (2.5%)	
White	108 (29.8%)	77 (21.3%)	
Gender, n (%)			0.122
Female	68 (18.2%)	53 (14.2%)	
Male	119 (31.8%)	134 (35.8%)	
Age, n (%)			0.069
<=60	79 (21.2%)	98 (26.3%)	
> 60	107 (28.7%)	89 (23.9%)	
Adjacent hepatic tissue inflammation, n (%)			0.490
None	68 (28.7%)	50 (21.1%)	
Mild	51 (21.5%)	50 (21.1%)	
Severe	11 (4.6%)	7 (3%)	
Vascular invasion, n (%)			0.249
No	112 (35.2%)	96 (30.2%)	
Yes	51 (16%)	59 (18.6%)	
OS event, n (%)			0.003
Alive	136 (36.4%)	108 (28.9%)	
Dead	51 (13.6%)	79 (21.1%)	
Age, median (IQR)	62 (52, 69)	60 (51, 68)	0.259

**Fig. 5:**
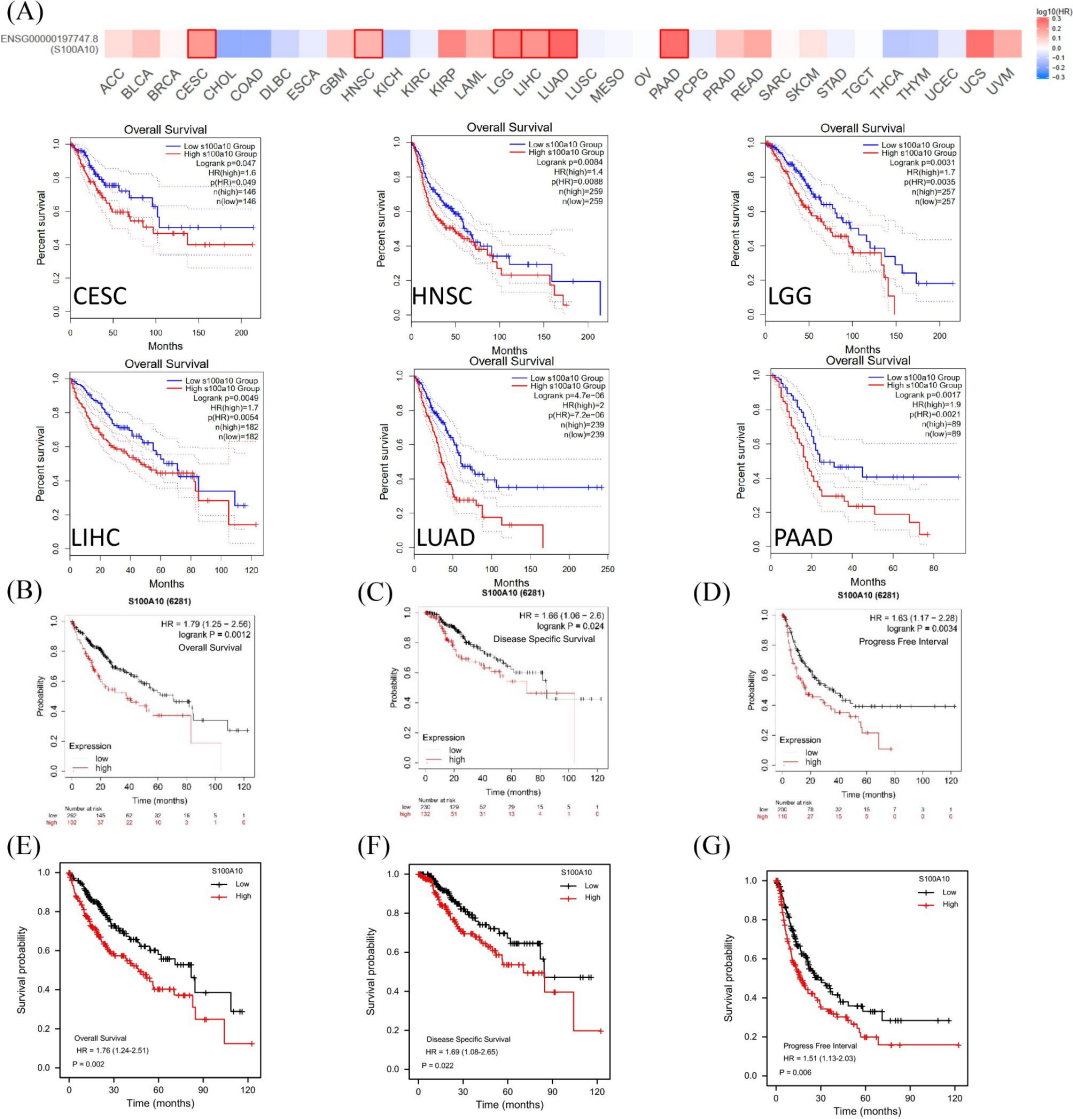
Kaplan-Meier survival curve analysis of the prognostic significance of S100A10 expression in different types of human cancers. **(A)** The correlation between S100A10 expression levels and OS in different tumors were analyzed using GEPIA2 website, TGCA database and GTEx database. **(B-D)** Kaplan-meier Plotter was used to analyze the expression levels of S100A10 in GEO, EGA and TCGA databases and there was a negative correlation with OS, DSS and PFI of HCC. **(E-G)** The expression level of VEGFA was negatively correlated with OS, DSS and PFI of HCC by TCGA database

Moreover, we drew the KM survival curve based on the data of GEPIA2.0 website, and found that the overall survival (OS) of the group with high S100A10 mRNA expression in CESC, HNSC, LGG, LIHC, LUAD and PAAD was lower than that of the group with low S100A10 mRNA expression (Fig. 5A). We further obtained HCC survival curves related to S100A10 through Kaplan Meier plotter website, and the results were consistent with TCGA results (OS (*e* = 0.0012), DSS(*e* = 0.024), PFI(*P* = 0.0034)) (Fig. 5B-D). Subsequently, we obtained the same result through TCGA database analysis. OS (*P* = 0.002), DSS (*P* = 0.022), PFI (*P* = 0.006)) (Fig. 5E-G). It has been confirmed in multiple databases that high expression level of S100A10 is associated with poor prognosis of HCC. We hypothesized that the prognostic correlation was related to the level of immune cell infiltration.

### S100A is related to tumor immune cell infiltration and immune microenvironment

**Fig. 6:**
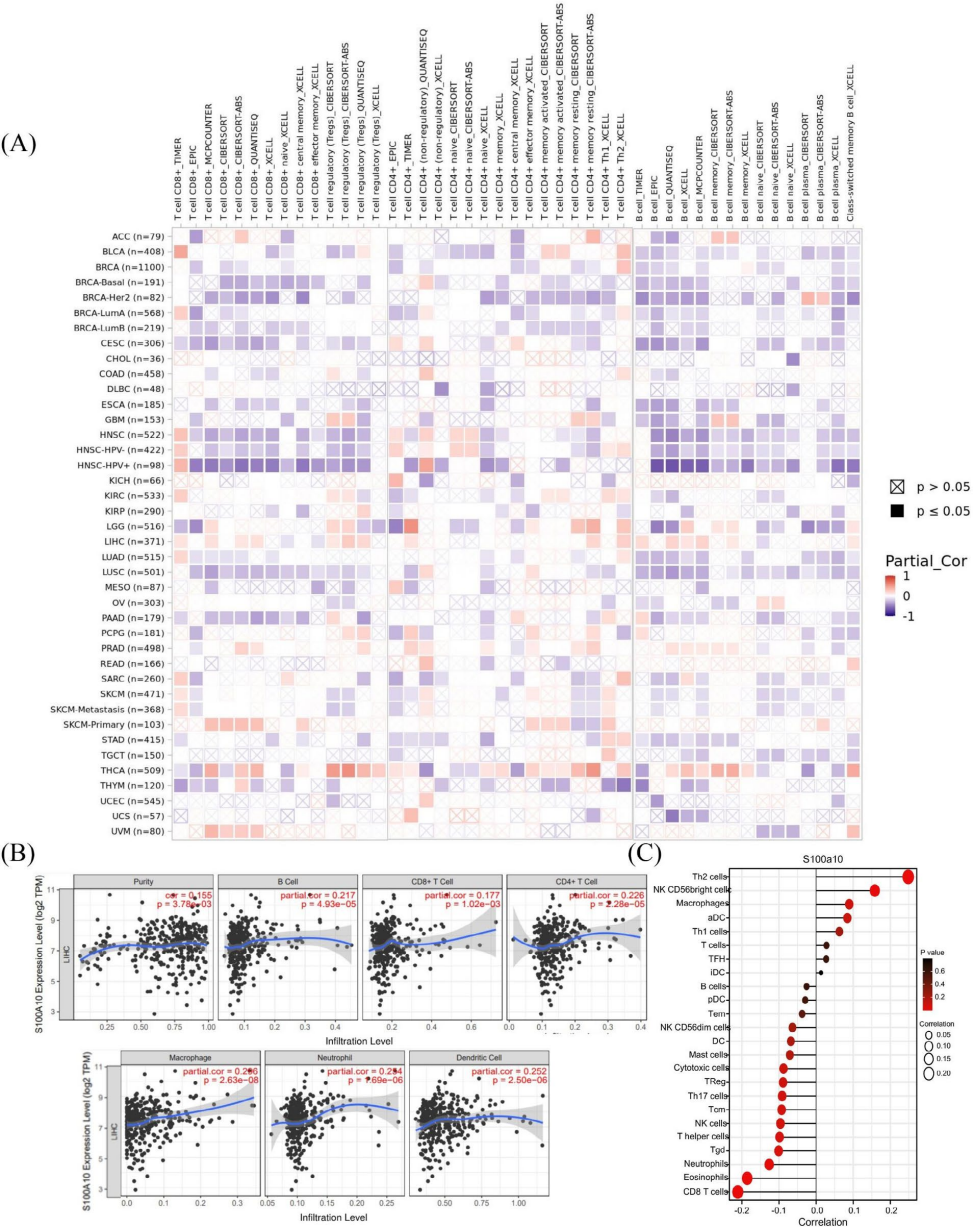
Correlation analysis of S100A10 expression and infiltration levels of immune cells in tumor tissues. **(A)** The relationship between S100A10 mRNA expression and CD8 + T cell, CD4 + T cell and Regulatory T Cell in various tumors was evaluated using a variety of algorithms based on the TIMER database. **(B)** S100A10 expression was positively correlated with tumor purity and infiltration levels of B cells, CD8 + T cells, CD4 + T cells, macrophages, and DCs in HCC tissues based on the TIMER database. **(C)** Correlation between S100A10 expression level and infiltration of various immune cells (Th2 Cell、NK CD56bright Cell、Macrophages、aDC、Th1 cell、TFH、iDC、B cell、pDC、Mast cell、Cytotoxic Cell、Tcm、NK Cell、T helper Cell、Tgd、Neutrophils、Eosinophils、CD8 + T cell) in HCC tissues

It is well known that tumor-infiltrating lymphocytes (TILs) influence the development of human tumors and affect the survival time of cancer patients. we used many algorithms to evaluate the relationship between S100A10 expression and the immune cells including CD8 + T cells, CD4 + T cells and regulatory T cells infiltration in various tumors (Fig. 6A). the results showed that S100A10 was related to tumor immune cell infiltration in many types of cancers including HCC. Next, we used the TIMER database to analyze the correlation between the expression of S100A10 and tumor purity and the infiltration of CD8 + T cells, CD4 + T cells, B cells, dendritic cells, macrophages and neutrophils. The results showed that the high expression of s100a10 was significantly positively correlated with the high infiltration of the above six immune cells. Among them, it was correlated with B cell (COR = 0.217, *P* < 0.001), CD8 + T cell (COR = 0.177, P < 0.001), CD4 + T cell (COR = 0.226, *e* < 0.001), macrophage (COR = 0.296, P < 0.001), neutrophil (COR = 0.254, *P* < 0.001), dendritic cell (COR = 0.252, *P* < 0.001). There was a similar correlation with tumor purity (COR = 0.155, *P* < 0.001) (Fig. 6B). In order to further study the infiltration of various immune cells in HCC tissues, we obtained from TCGA database that Th2 cells, T helper cells, TFH, ADC, NK CD56 bright cells, macrophages, T cells, etc. have a high correlation between immune infiltration and S100As expression. Among them, the infiltration level of Th2 cells was positively correlated with the expression level of S100A10 mRNA, while the infiltration level of CD8 + T cells was negatively correlated with the expression level of S100A10 mRNA. (Fig. 6C).

### Network enrichment analysis identifies S100A10 functions, associated signaling pathways and genes

**Fig. 7:**
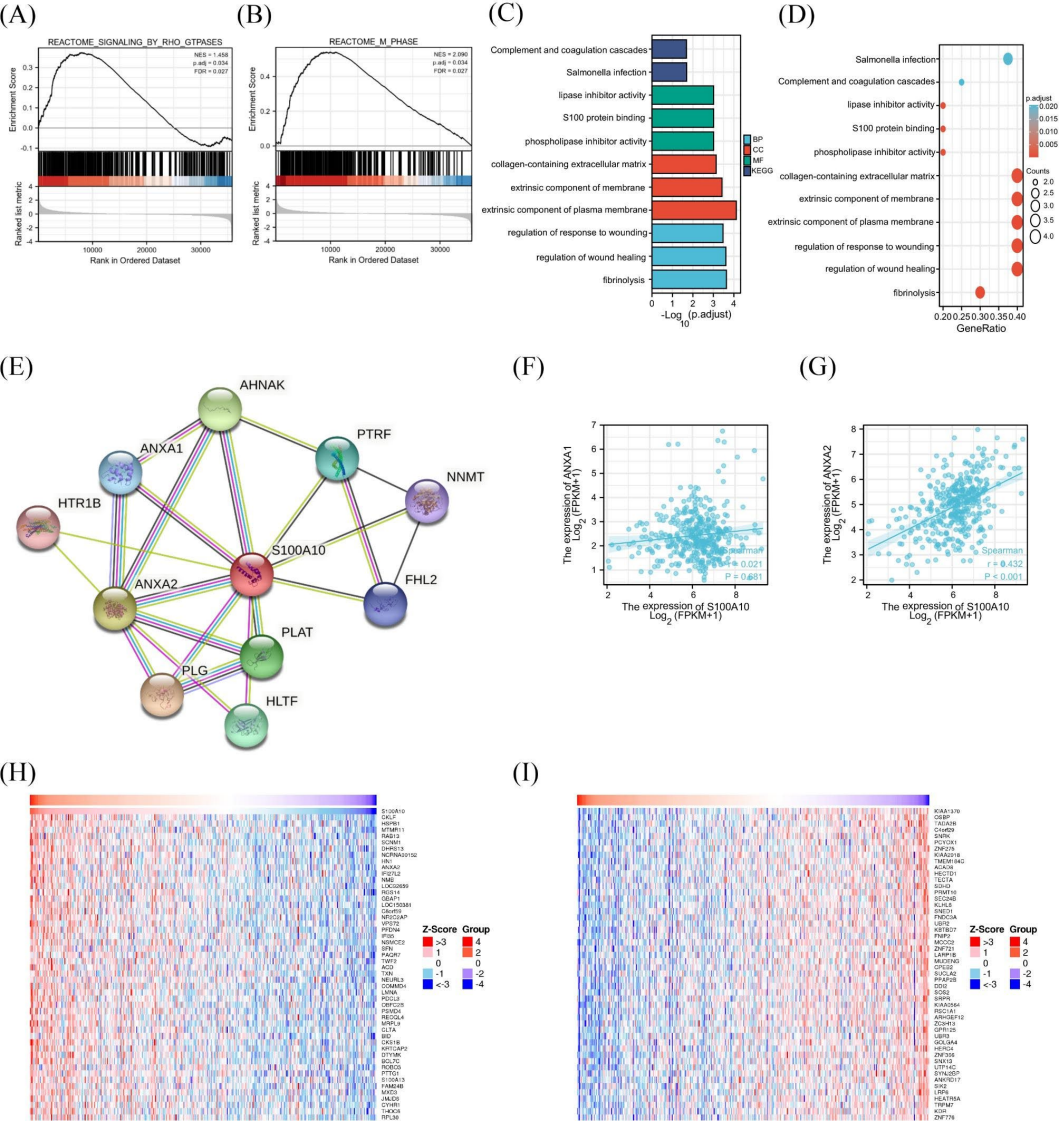
Enrichment analysis of S100A10 functional networks. **(A-B)** Enrichment plots by GSEA. **(C-D)** Enrichment of gene ontology (GO) terms and Kyoto Encyclopedia of Genes and Genomes (KEGG) for genes related to S100A10. **(E)** Protein–protein interaction network of S100A10. **(F-I)** Correlation between S100A10 expression levels and ANXA1 and ANXA2 expression levels. **(J)** The heat map shows the top 50 genes positively related to S100A10 in the HCC cohort. **(K)** The heat map shows the top 50 genes negatively related to S100A10 in the HCC cohort

S100A10 activated two pathways: reaction signaling by Rho GTPases and reactome M phase (Fig. 7A, B). GO and KEGG analysis showed 3 signal pathways (BP), 3 cell components (CC), 3 biological processes (MF), and 2 KEGG respectively. We found that S100A10 was involved in the lipase inhibitor activity, S100 protein binding, phospholipase inhibitor activity, collagen-containing extracellular matrix, an extrinsic component of membrane, extrinsic component of the plasma membrane, regulation of response to wounding, regulation of wound healing and fibrinolysis, *.etc* by GO analysis. While, KEGG analysis showed that S100A10 was involved in complement and coagulation cascades and salmonella infection (Fig. 7C, D).

Protein interactions coordinate various biological functions, and the study of protein interactions is crucial for understanding the molecular mechanisms of biological processes and pathophysiology [19]17](Yang et al., 2017)(Yang, et al., 2017) [17][Yang et al., 2017][Yang et al., 2017]^17^ [17](Yang et al., 2017) [17] [17]. In order to explore the interaction between proteins in HCC, we obtained the interaction network diagram between proteins related to S100A10 from the STRING website (Fig. 7E). Among them, there is a strong correlation between ANXA1, ANXA2 genes and the expression level of S100A10. (Fig. 7F, G). In addition, the TOP 50 genes positively and negatively correlated with s100aF gene expression were also exhibited by the heat map (Fig. 7H, I). Next, we identify the S100A10 associated signaling pathways and genes in L02 cell lines (use knock out; showed in WB.) All the results provided new information for the in-depth understanding of S100A.

### Inhibiting S100A10 reduces the proliferation, invasion and migration of HCC cells and promotes apoptosis

**Fig. 8:**
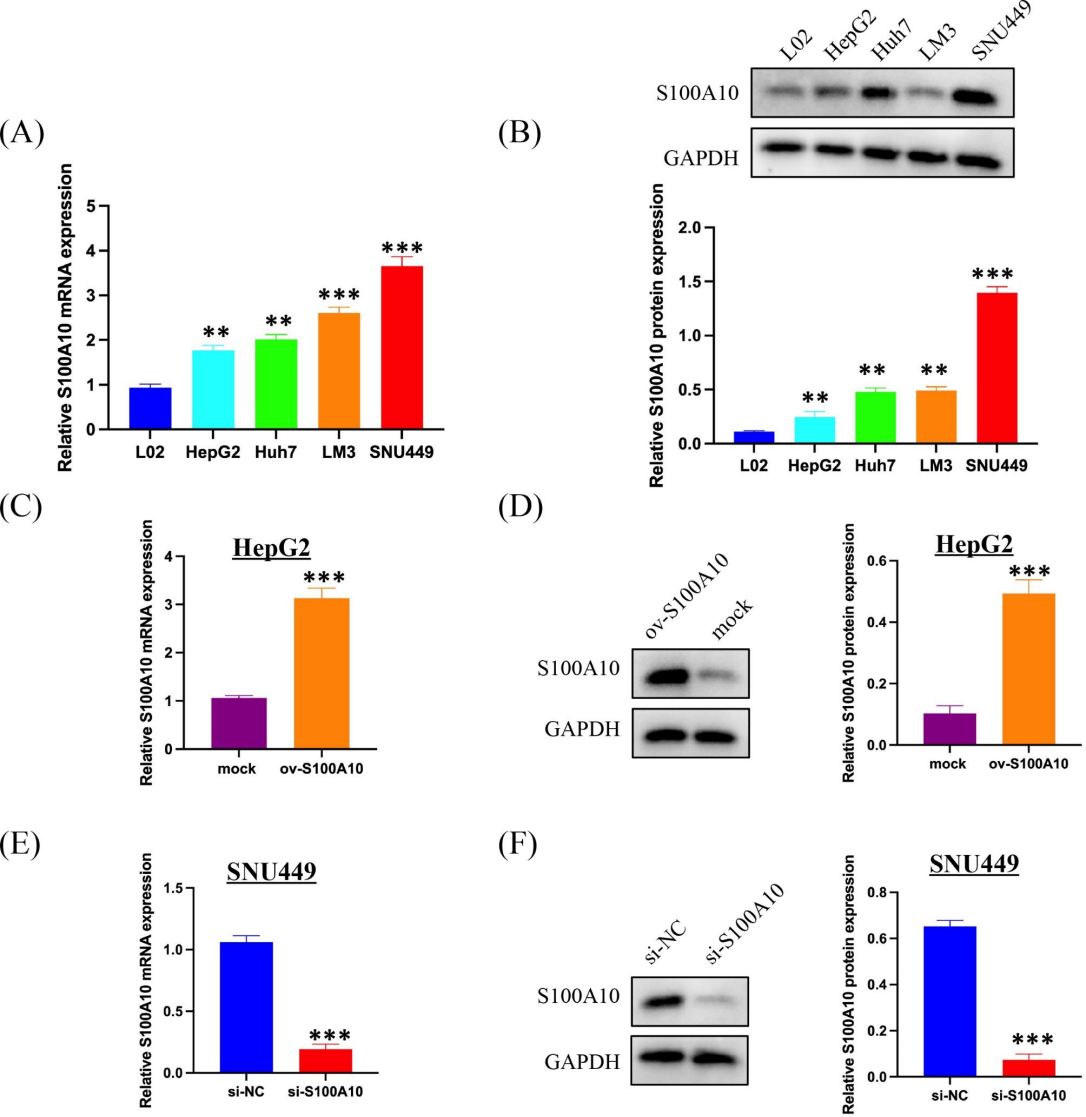
Expression level of S100A10 in hepatocytes and hepatocellular carcinoma cell lines. **(A)** Expression of S100A10 mRNA in hepatocytes (L02) and hepatocellular carcinoma cell lines (HepG2、Huh7、LM3、SNU449) by PCR. **(B)** Expression of S100A10 protein in hepatocytes (L02) and hepatocellular carcinoma cell lines (HepG2、Huh7、LM3、SNU449) by Western Blot. **(C-D)** Overexpression efficiency of S100A10 in HepG2. **(E-F)** S100A10 SNU449 knockdown efficiency. Note: **P < 0.01, ***P < 0.001

**Fig. 9:**
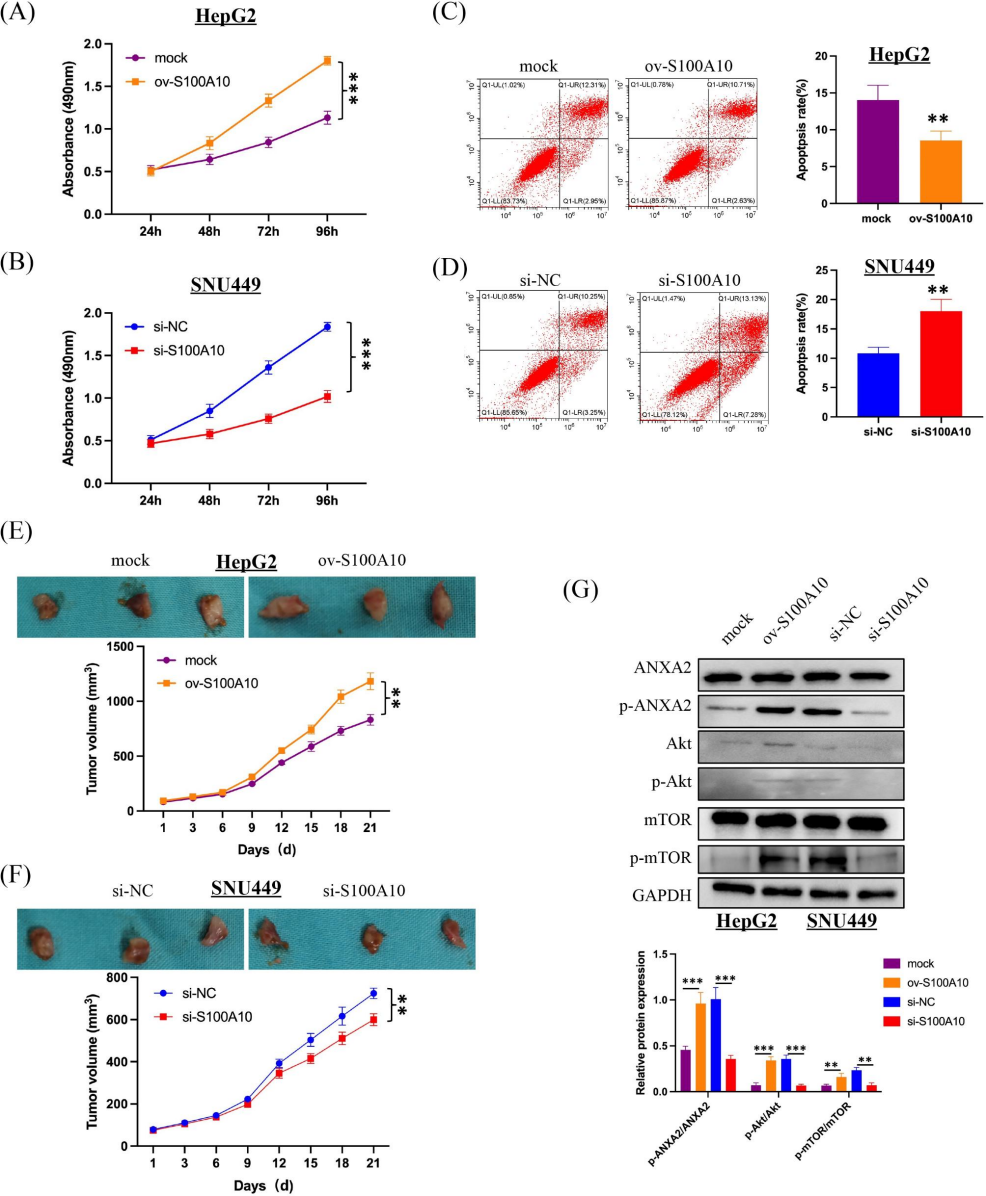
Inhibition of S100A10 reduced the proliferation, invasion and migration of HCC and promoted apoptosis. **(A-B)** The proliferation ability of HCC cells was positively correlated with the expression level of S100A10 by CCK8 assay. **(C-D)** The expression level of S100A10 was negatively correlated with the apoptosis level of HCC cells. **(E-F)** S100A10 promoted tumor growth in nude mice. **(G)** Expression and phosphorylation of S100A10 downstream effector gene (ANXA2、Akt、mTOR) in nude mouse tumor tissue. Note: **P < 0.01, ***P < 0.001

To further verify the biological functions of S100A10, we examined the expression levels of S100A10 mRNA and protein in hepatocytes (L02) and hepatoma cells (HepG2, Huh7, LM3, SNU449) (Fig. 8A, B). In hepatocellular carcinoma cells, S100A10 expression was lowest in HepG2 and highest in SNU449. Therefore, we established HepG2 cells that stably overexpressed S100A10 (ov-S100A10) and SNU449 cells that stably silence expressed S100A10(si-S100A10) (Fig. 8C-F). By CCK8 assay we found that the cell viability was higher for ov-S100A10 and lower for si-S100A10(Fig. 9A, B). Overexpression of S100A10 reduced apoptosis of hepatocellular carcinoma cells, while silencing of S100A10 increased apoptosis of hepatocellular carcinoma cells (Fig. 9C, D). To further explore the role of S100A10 in vivo, we grew HepG2 cells with ov-S100A10 and SNU449 cells with si-S100A10 subcutaneously in nude mice. On the premise of successful tumor formation, tumor volume was observed every three days for 21 days. No matter HepG2 or SNU449, the growth rate of the tumor was positively correlated with the expression level of S100A10 (Fig. 9E, F). S100A10 regulates apoptosis and viability of hepatocellular carcinoma cells probably associated with ANXA2/Akt/mTOR pathway [15, 20–22]. We found that the expression level of S100A10 was positively correlated with the phosphorylation levels of ANXA2, Akt, and mTOR by Western Blot (Fig. 9G). In summary, we basically concluded that S100A10 was involved in the growth process of hepatocellular carcinoma through the ANXA2/Akt/mTOR signaling pathway.

## Discussion

HCC ranks third in cancer-related death worldwide owing to its high incidence and linked mortality [23]. Although advances in the knowledge of HCC biology and therapy, the fatality if this disease is still high. Evidences indicated that the precise identification and differential diagnosis of early HCC can significantly increase the survival of patients. In the present study, we comprehensively analyzed the role of S100As in HCC including expression, mutation, prognostic value and immune cells infiltration.S100As proteins, small calcium-binding dimeric proteins, were originally shown to be found in vertebrates exclusively. S100As proteins, encoded by *S100As* genes which are located on chromosome 1q21 in humans in the district of the epidermal differentiation complex, are high structural homology and pleiotropic function [7]. According to reports, it ultimately leads to apoptosis, glycogen phosphorylation, phagocyte recruitment, and so on through involving in the activation of particular biochemical ways [24, 25]. The S100A family members have been demonstrated to play an important role in the interaction between tumor cells and their micro-environment, even involving the process of malignant tumor metastasis [26, 27]. Many studies have shown that S100A10 is involved in the occurrence and development of ovarian, lung, colorectal and pancreatic cancers [10, 13, 22, 28–30]. Even with evidence that S100As have been confirmed to be associated with the development of many digestive cancers, distinct roles of S100As in HCC remained to be investigated. Furthermore, the function of S100A10 and related mechanisms were verified by the experimental analysis.

The TCGA database was applied to analyze the differences between expression of S100As in LIHC samples and normal samples. We learned that mRNA expression levels of S100A4, S100A6, S100A10, S100A11 and S100A16 were higher in HCC than in normal tissues. It Studies have shown that multiple members of the S100As are abnormally expressed in tumors and are widely involved in processes related to tumorigenesis and progression [22, 23]. In order to indicated the role of the family members in HCC development, we further analyzed the expression of S100A4, S100A6, S100A10, S100A11 and S100A16 in pan-cancer, and the results suggested that they were highly expressed in most tumors. Furthermore, their protein expression was analyzed by the CTPAC database, and only S100A6 and S100A10 protein expression was different, and S100A6 protein expression was lower in tumors than in normal tissues, the protein expression level of S100A10 in HCC was higher than that of normal tissues. Our results illustrated that the mRNA expression of S100A6 and protein expression of S100A6 were inconsistent. The expression of mRNA does not necessarily have a linear relation to its translation product-protein expression, because there are several levels of regulation of gene expression, and the regulation of transcription level is only one connection, and post-transcriptional regulation and translation and post-translational regulation are all related to the expression of the final protein. There are many factors that may cause the expression levels of mRNA and protein to be inconsistent, including mRNA degradation, protein degradation, modified folding and so on. This may be caused by the rapid degradation of mRNA expression of S1006 in LIHC tissues, or the slow degradation of protein expression of S1006. Although some reports also evidenced that S100A10 participated in the development of ovarian cancer, breast cancer, and gastric cancer via different pathways, the mechanism for the function of S100A10 has not been clarified. We further analyzed the role and underlying mechanism of S100A10 in the development of HCC [31].

Using a large amount of data from multiple public databases, we concluded that high expression of S100A10 is associated with a poor prognosis of HCC. This conclusion may be related to immune cell infiltration. Early in 1985, Virchow had put forward this point of view [32]. Early in the initial stage, the inflammatory cells in different types of human tumors are powerful tumor promoters33 [2]. As cancers develop, the presence of proliferating inflammatory cells plays an important role in tumor cell migration, invasion and poor clinical outcomes [32, 33]. The S100A10 was related to the most infiltration status of immunocytes. In addition, through functional enrichment analysis, we found that S100A10 activates reaction signaling by Rho GTPases and Reactome M phase signal pathways and participates in biological functions such as complement and coagulation cascades and salmonella infection.We used TIMER to study the potential relationship between different immune cell infiltration levels and S100A10 in LIHC. This results was similar to these previous studies, suggesting that the S100A10 may be a potential target for the immunotherapy of HCC. However, the specific mechanism should be further investigated.

A large number of studies have shown that ANXA2 is involved in the development of hepatocellular carcinoma and plays an important role in its growth, and high expression of ANXA2 is negatively correlated with the prognosis of hepatocellular carcinoma [34–36]. ANXA2 interacts with S100A10 in gastric cancer to activate the mTOR pathway and inhibit apoptosis in gastric cancer cells [15]. To further explore the biological function of S100A10 in HCC. We speculate that S100A10 and ANXA2 may also activate the Akt/mTOR pathway in hepatocellular carcinoma. Finally, we come to the conclusion that the expression level of S100A10 was positively correlated with the phosphorylation levels of ANXA2, Akt, and mTOR. Nevertheless, some limitations in our study should be recognized. In the further research, we will combine the public databases and HCC cohorts to study the function of S100As in HCC.

## Electronic supplementary material

Below is the link to the electronic supplementary material.

Supplementary Material 1

## Data Availability

The datasets used and/or analysed during the current study available from the corresponding author on reasonable request.
