# Supplementary material for: S100A family is a group of immune markers associated with poor prognosis and immune cell infiltration in hepatocellular carcinoma
Source: BMC Cancer. 2023 Jul 7;23:637. doi: 10.1186/s12885-023-11127-3 (PMC10327351; doi:10.1186/s12885-023-11127-3)

Figure3 S100A10

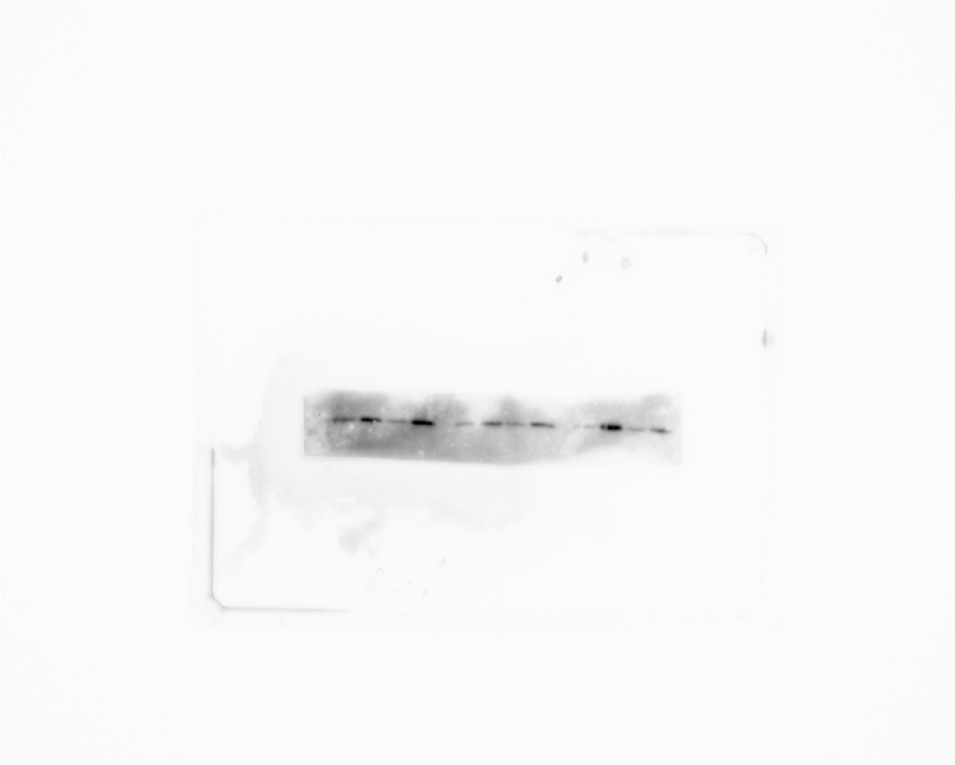

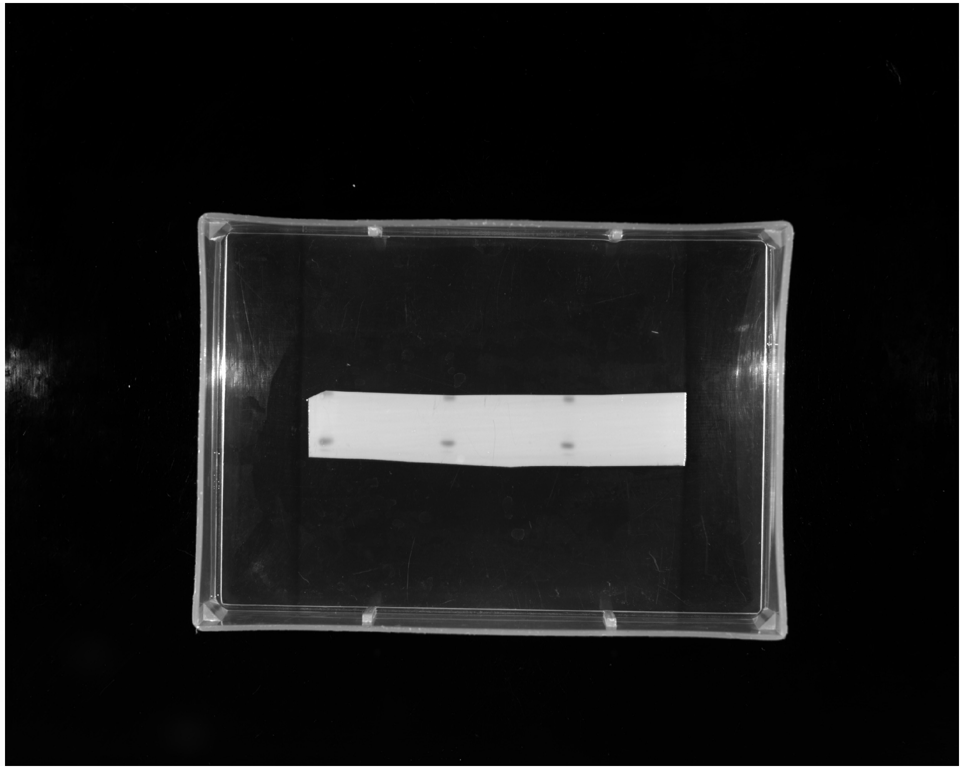

Figure3 GAPDH

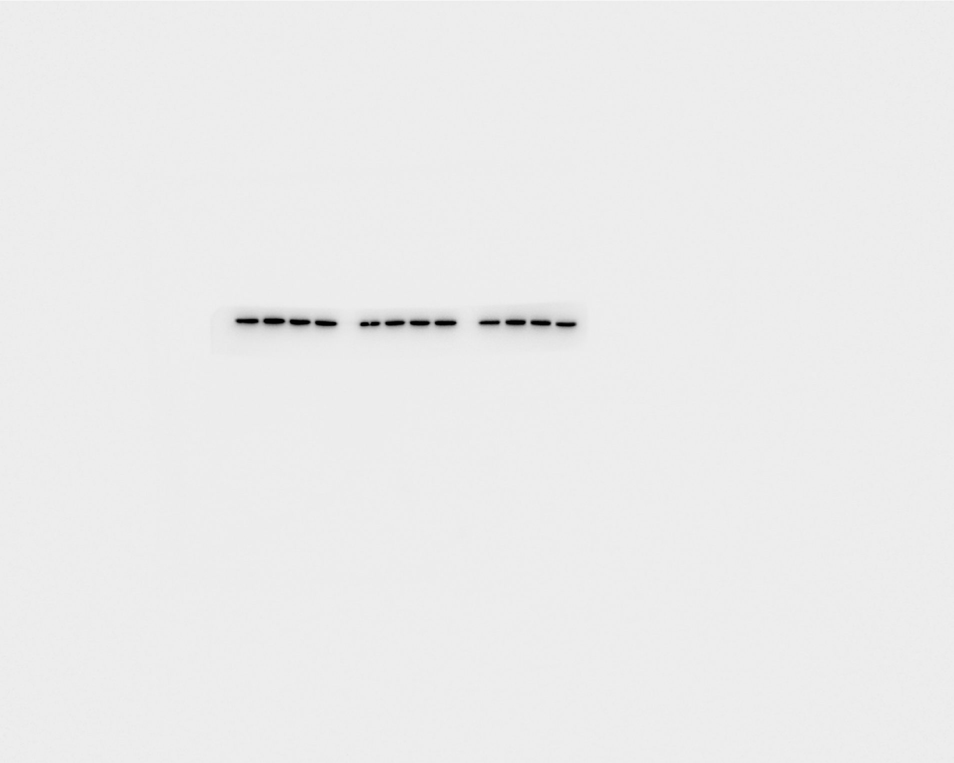

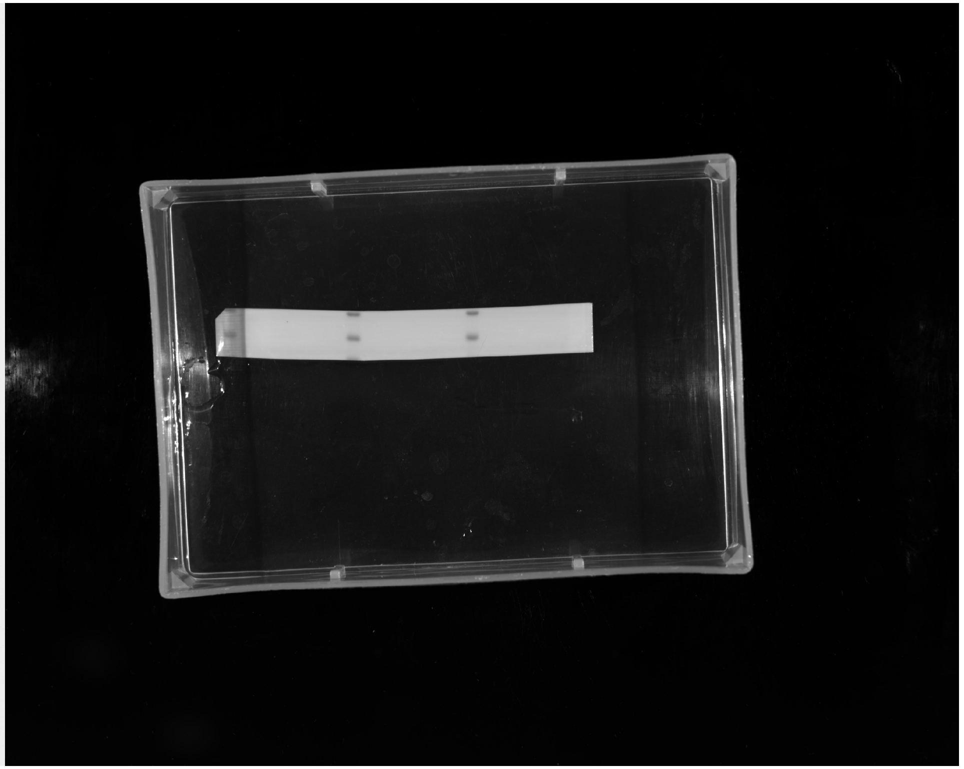

Figure8 S100A10

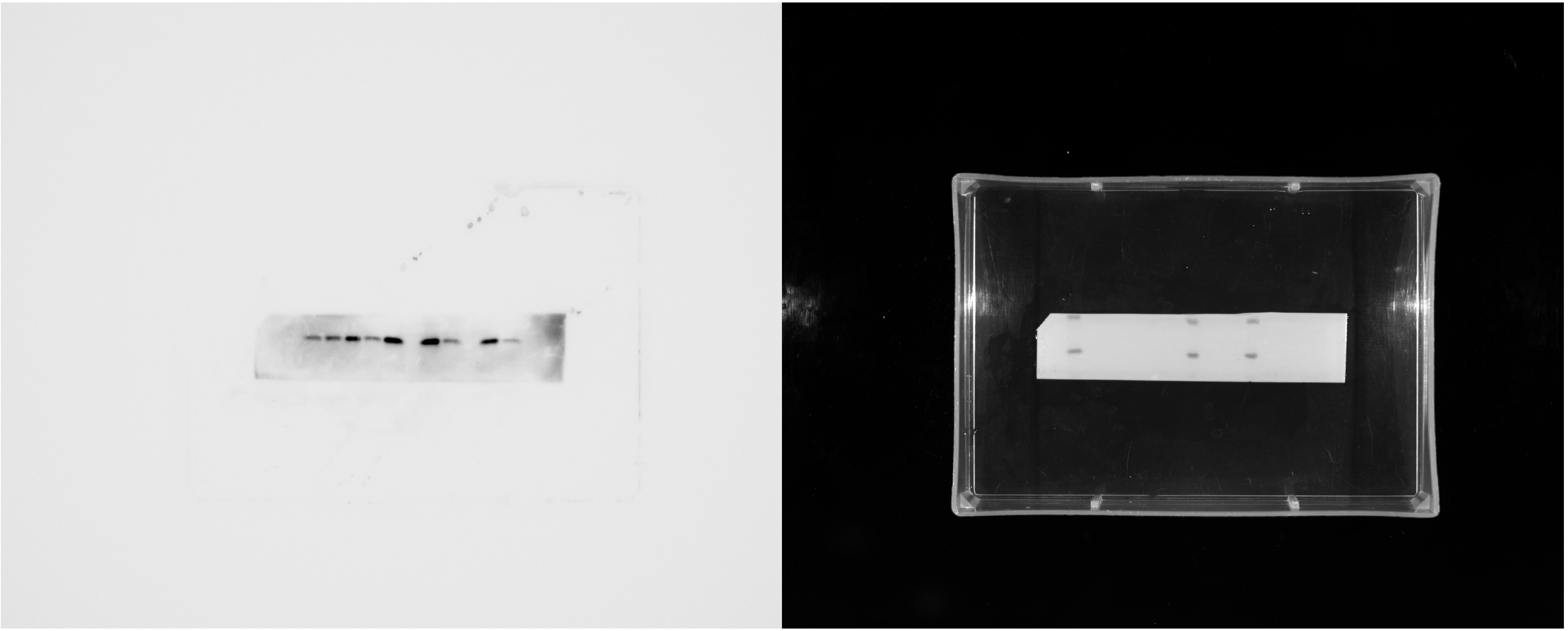

Figure8 GAPDH

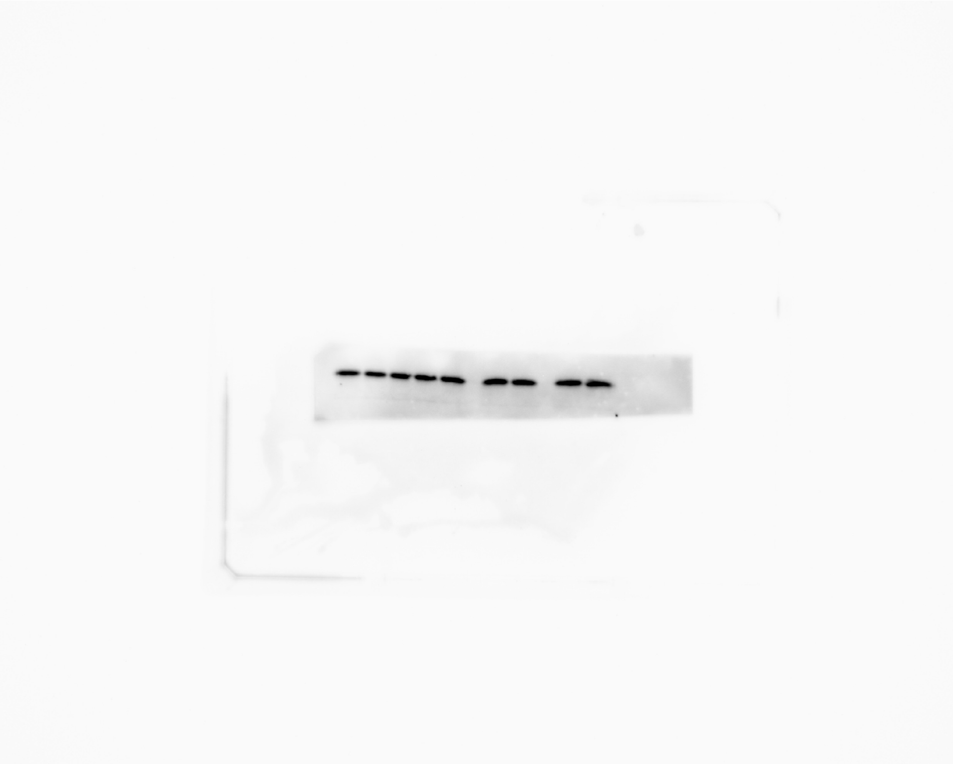

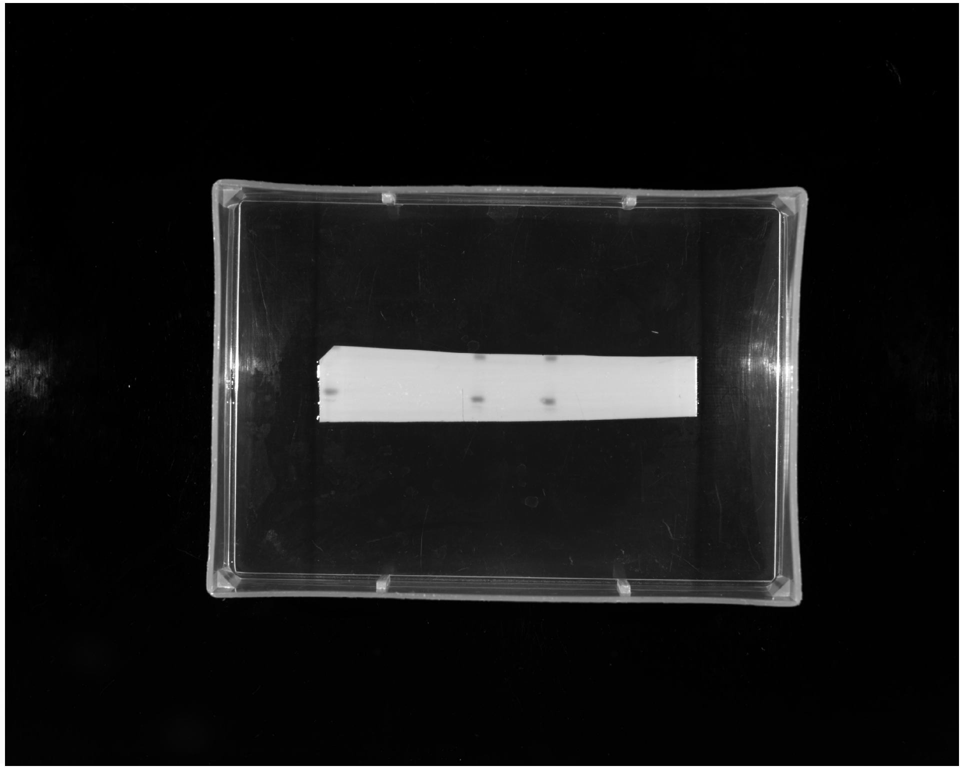

Figure9 ANXA2

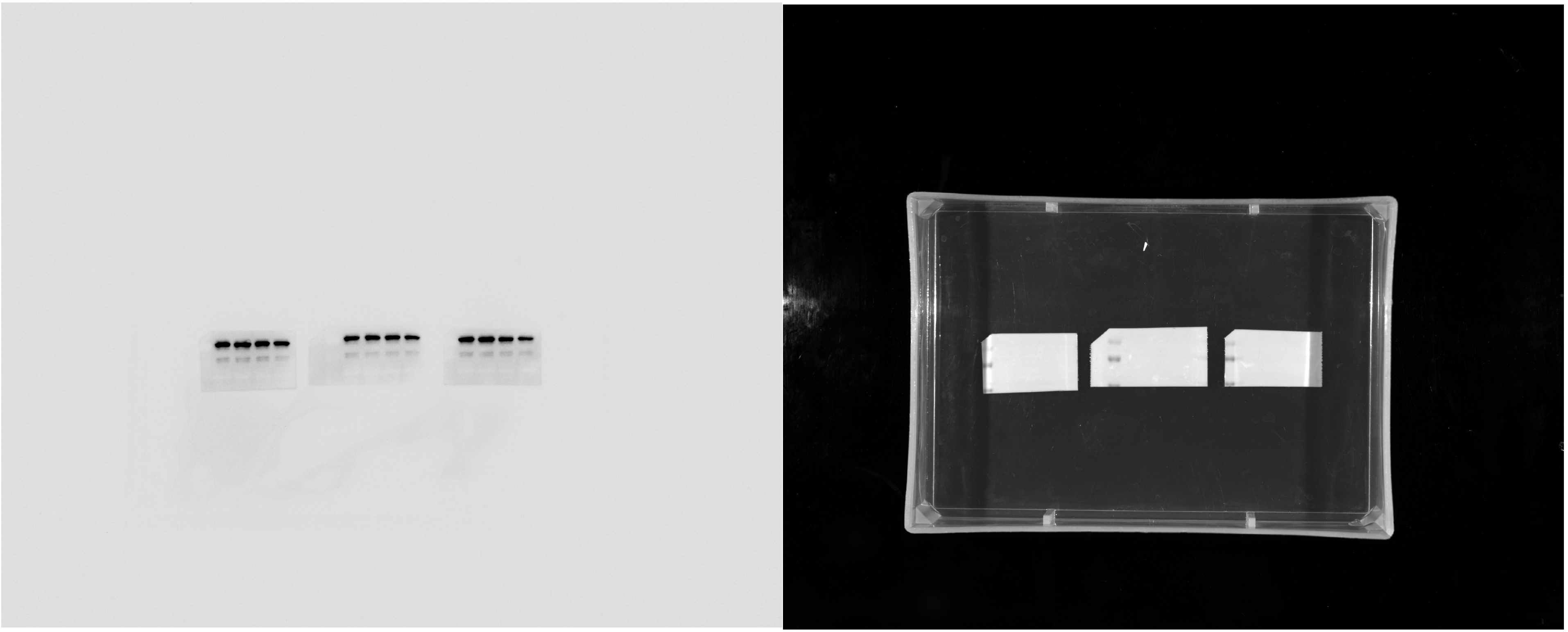

Figure9 p-ANXA2

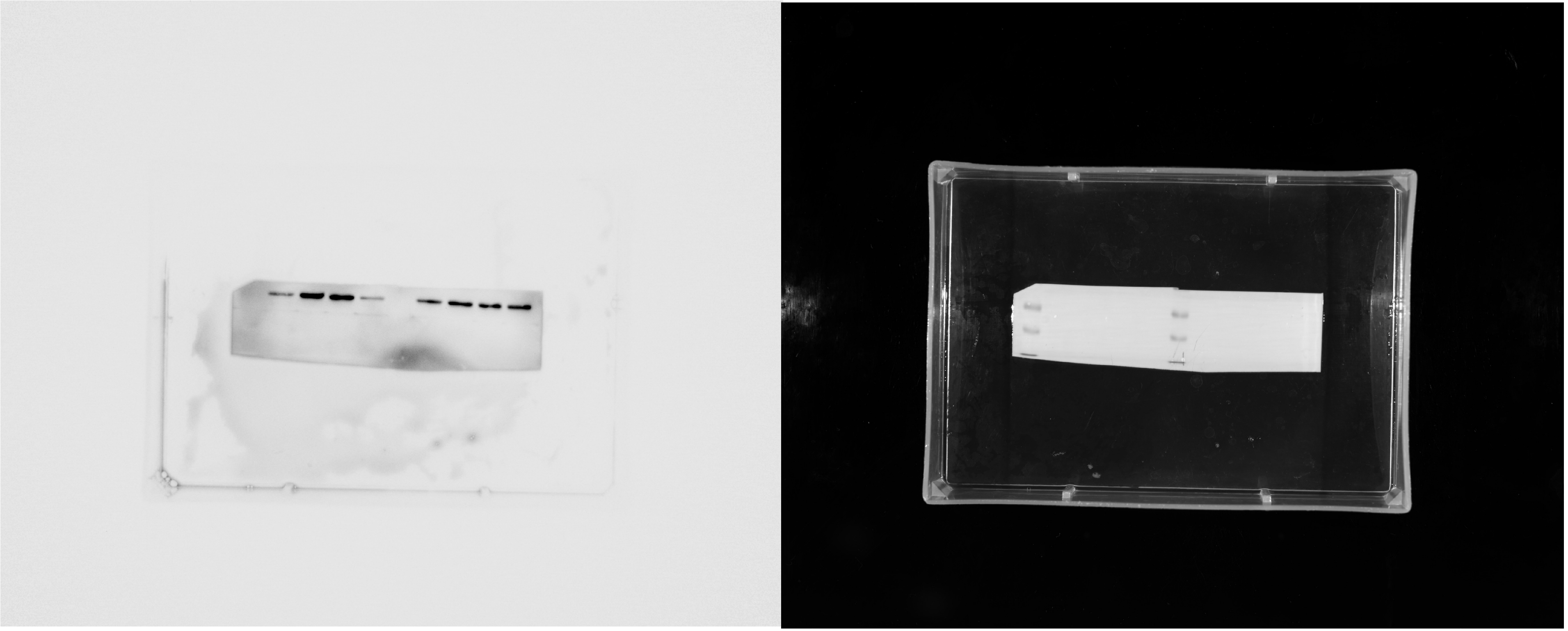

Figure9 Akt

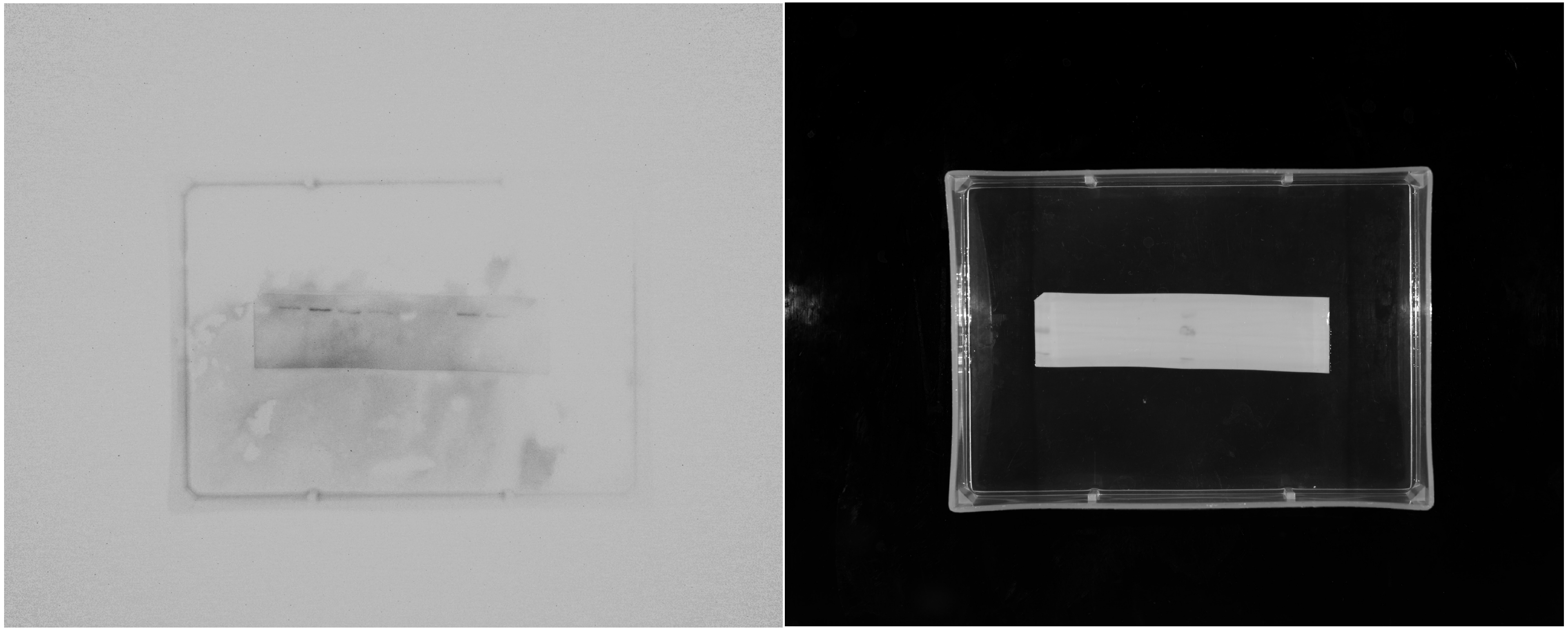

Figure9 p-Akt

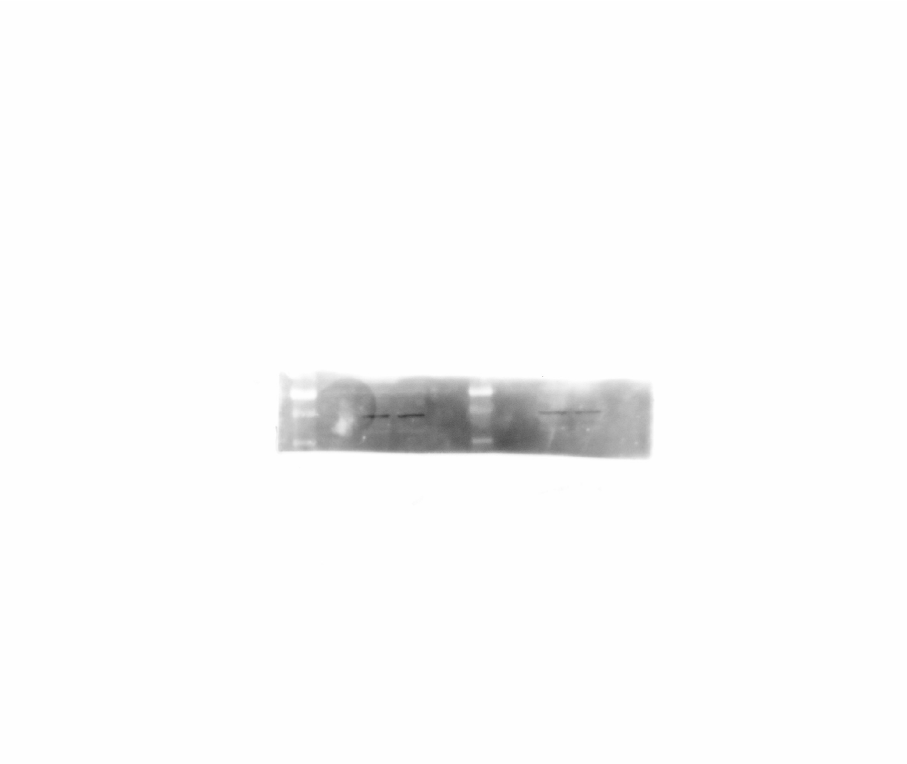

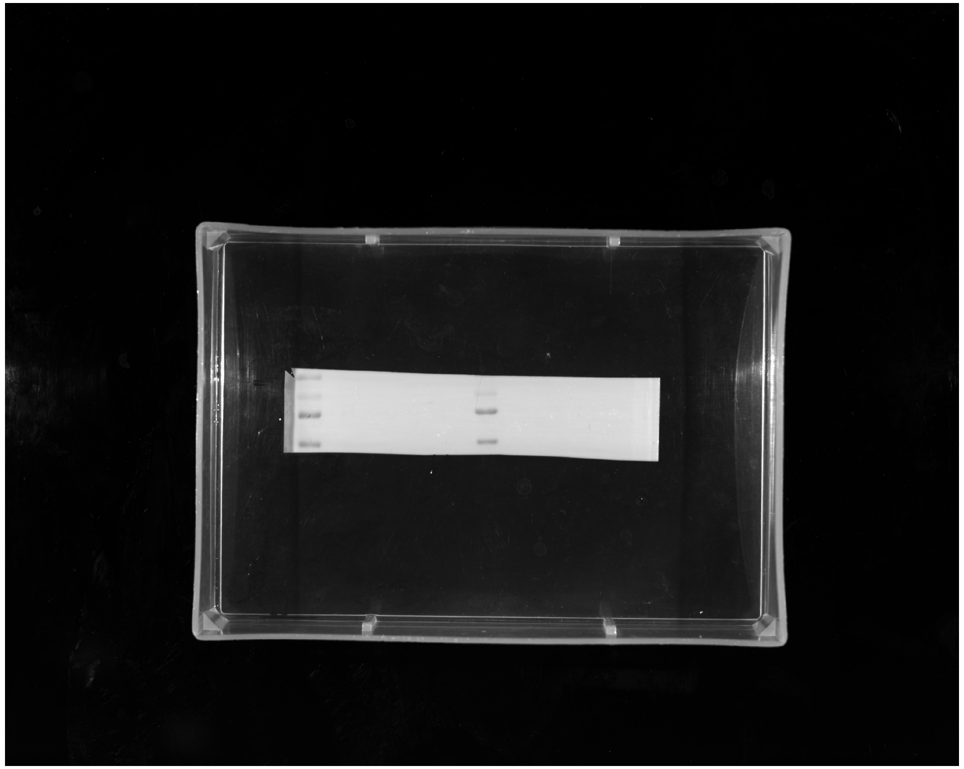

Figure9 mTOR

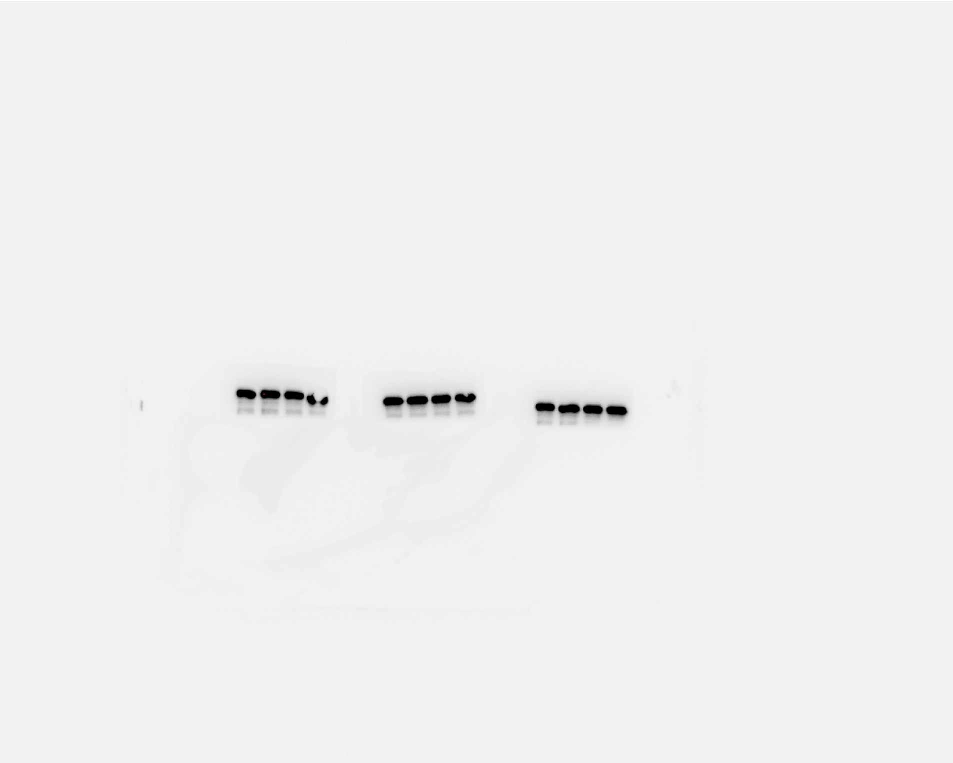

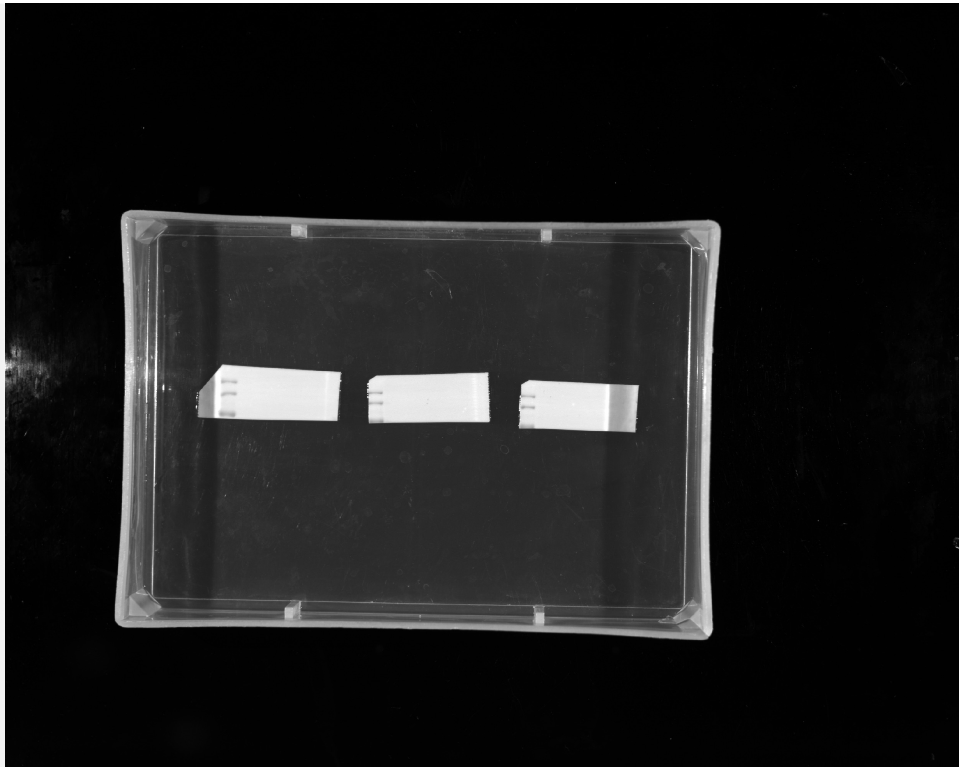

Figure9 p-mTOR

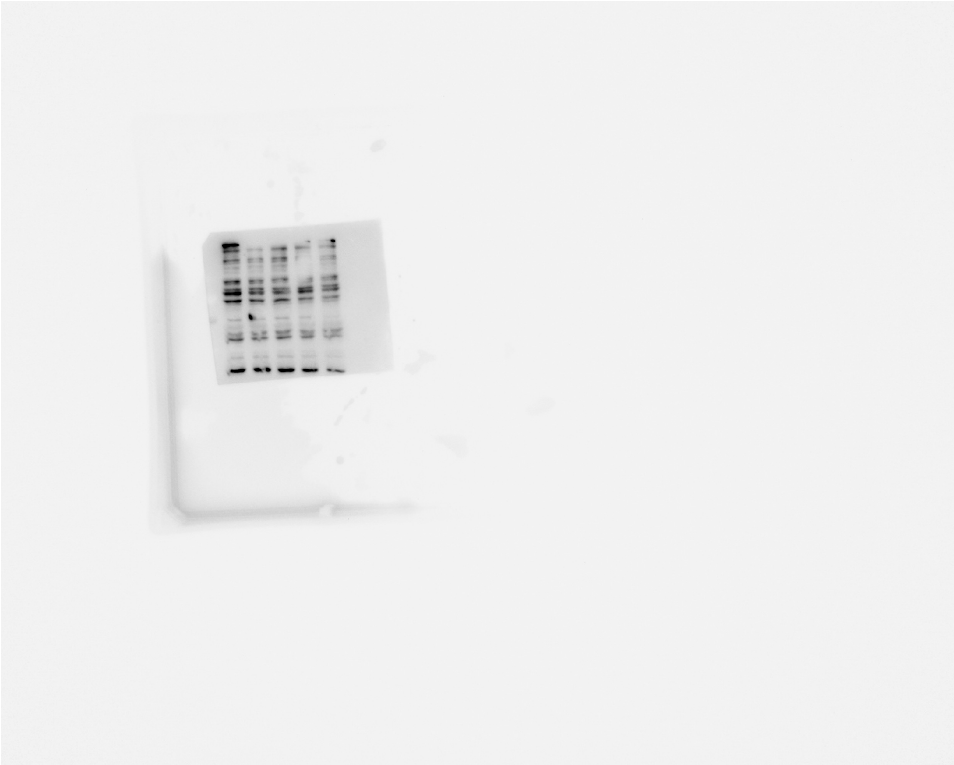

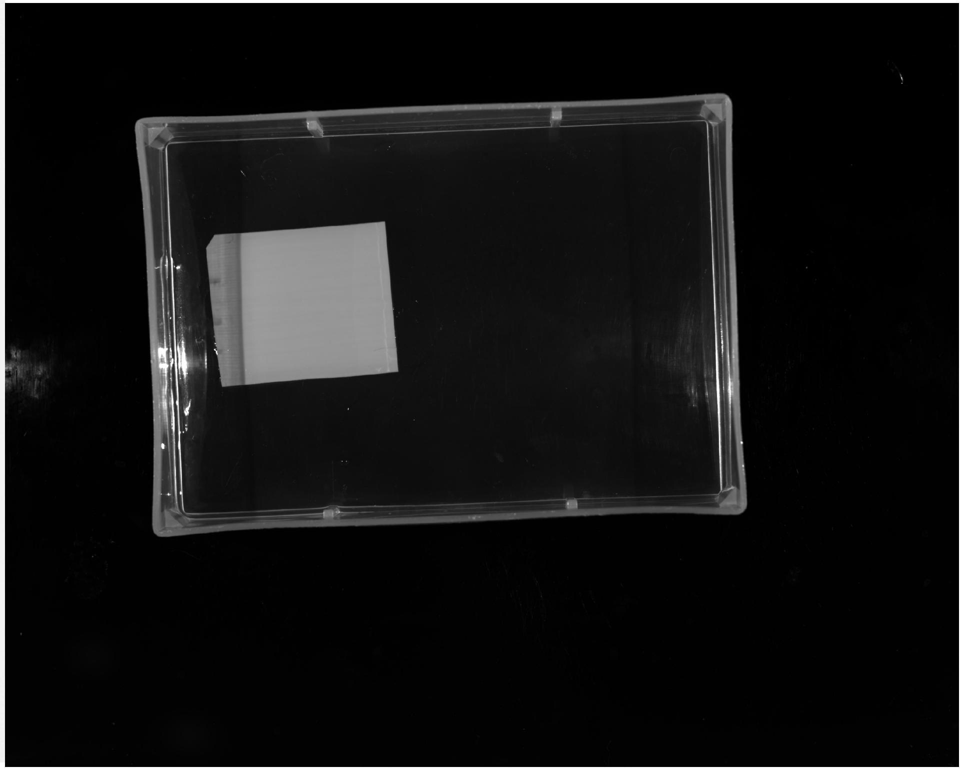

Figure9 GAPDH

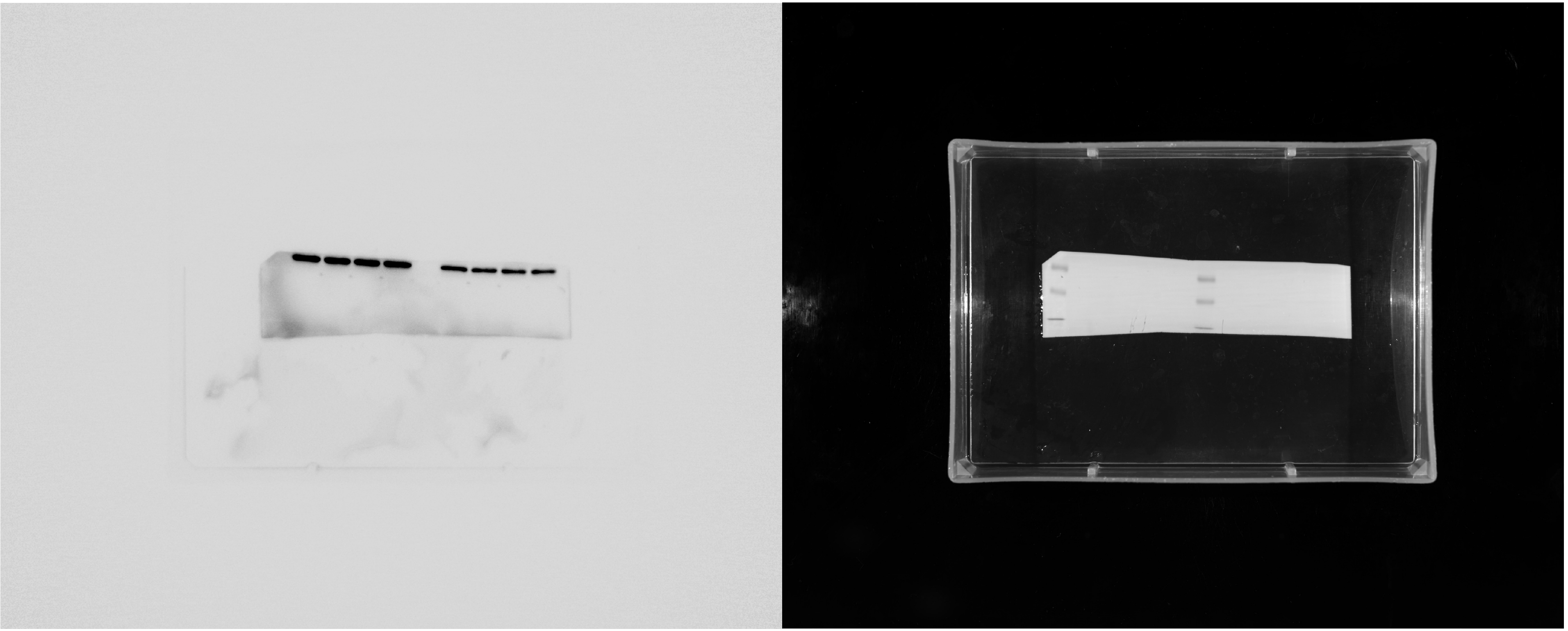

Supplement: Supplementary file 1 — Supplementary Material 1 [file 12885_2023_11127_MOESM1_ESM.pdf]
